# Selective sorting and secretion of hY4 RNA fragments into extracellular vesicles mediated by methylated YBX1 to promote lung cancer progression

**DOI:** 10.1186/s13046-022-02346-w

**Published:** 2022-04-11

**Authors:** Chuang Li, Wei Wang, Yuting Sun, Yifan Ni, Fang Qin, Xiaolu Li, Tao Wang, Mingxiong Guo, Guihong Sun

**Affiliations:** 1 School of Basic Medical Sciences, Wuhan University, Wuhan, Hubei P. R. China; 2 Hubei Key Laboratory of Cell Homeostasis, College of Life Sciences, Wuhan University, Wuhan, Hubei P. R. China; 3 Department of Radiation Oncology, Hubei Cancer Hospital, Tongji Medical College, Huazhong University of Science and Technology, Wuhan, Hubei P. R. China; 4 Department of Respiratory and Critical Care Medicine, Tongji Hospital, Tongji Medical College, Huazhong University of Science and Technology, Wuhan, Hubei P. R. China; 5 Hubei Provincial Key Laboratory of Allergy and Immunology, Wuhan, Hubei P. R. China

**Keywords:** Extracellular vesicles, hY4F, Non-small cell lung cancer, YBX1, Lysine methylation

## Abstract

**Background:**

Extracellular vesicles (EVs) are emerging mediators of intercellular communication that have been shown to play important roles in tumor progression. YRNA fragments, a type of small non-coding RNA, are dysregulated in non-small cell lung cancer (NSCLC) cell-derived EVs, suggesting that they may be an effective biomarker for cancer diagnosis and treatment strategies.

**Methods:**

Differentially expressed YRNA hY4 fragments (hY4F) in EVs from NSCLC cells and normal lung fibroblasts were isolated by differential ultra-centrifugation. RNA-binding proteins that interacted with hY4F were identified by screening with an RNA pulldown assay and mass spectrometry. The molecular mechanism of hY4F and the RNA-binding protein Y box binding protein 1 (YBX1) was demonstrated by qRT-PCR, western blot, RNA pulldown, and rescue experiments. Transcriptome sequencing, qRT-PCR validation, bioinformatics analysis and NF-κB pathway inhibitor assays elucidate the mechanism of YBX1 and hY4F inhibiting lung cancer. A peptide pulldown assay was performed to screen and identify a potential methyltransferase for YBX1. The roles of hY4F, YBX1, and SET domain containing 3 in biological functions, such as proliferation, migration, invasion, and apoptosis, in lung cancer cells were also examined by EdU incorporation assay, Transwell assay, flow cytometry, and other methods. Lastly, a mouse xenograft assay was used to assess the clinical relevance of YBX1 and hY4F in vivo.

**Results:**

Our data demonstrate that hY4 RNA fragments were upregulated in lung cancer- derived EVs, hY4F inhibits tumor progression through downregulating MAPK/NF-κB signaling, and then the selective sorting and secretion of hY4F into lung cancer EVs is regulated by the RNA-binding protein YBX1. Furthermore, we identified lysine K264 within the YBX1 C-terminal domain as the necessary site for its interaction with hY4Fs. K264 is modified by methylation, which affects its binding to hY4F and subsequent selective sorting into EVs in lung cancer cells.

**Conclusion:**

Our findings demonstrate that hY4F acts as a tumor suppressor and is selectively sorted into lung cancer cell-derived EVs by interacting with methylated YBX1, which in turn promotes lung cancer progression. hY4F is a promising circulating biomarker for non-small cell lung cancer diagnosis and prognosis and an exceptional candidate for further therapeutic exploration.

**Supplementary Information:**

The online version contains supplementary material available at 10.1186/s13046-022-02346-w.

## Introduction

Extracellular vesicles (EVs) are double-membrane-bound vesicles with a diameter of 30 to 1000 nm that are either shed from the cell membrane or secreted [1]. EVs are mainly composed of micro-vesicles and exosomes. Micro-vesicles are vesicles that detach from the cell membrane and have a diameter of about 100 to 1000 nm [2]. Exosomes, which have a diameter of about 40 to 150 nm, are generated first from late endosomes and then fuse with multivesicular bodies and are released from the cell membrane [3]. Many studies have demonstrated that EVs play important roles on cancer genesis, progression, and metastasis by delivering cargo, such as proteins, lipids, DNA, and RNA, between different cells [2]. RNA can be sorted into EVs, protected from RNase degradation, and internalized by neighboring or distant cells, where they subsequently modulate biological processes [4].

YRNAs are 84 to 113 nucleotide small non-coding RNAs (ncRNA) with stem-loop structures that bind to Ro60 protein to form Ro ribonucleoproteins (RoRNPs) [5]. YRNAs is thought to be involved in initiating DNA replication and RNA quality control [6, 7]. Four YRNAs exist in humans: hY1, hY3, hY4, and hY5. YRNAs are upregulated in some human tumor tissues, and hY1 and hY3 are required for cell proliferation [8], suggesting that YRNAs are closely associated with carcinogenesis. YRNA fragments have been found in EVs from cancer cells [9] and human blood [10], suggesting a potential function in EVs-mediated intercellular communication. Although previous studies demonstrated that YRNAs and YRNA-derived small RNAs may play a role in carcinogenesis due to their altered expression in cancers, knowledge of their functions remains limited.

Lung cancer is globally the most commonly diagnosed cancer (11.6% of total cases) and the leading cause of cancer death (18.4% of total cancer deaths) in both sexes combined [11–13]. In recent years, many studies have demonstrated that EVs-derived ncRNAs are closely related to lung cancer progression. For example, hypoxic bone marrow-derived mesenchymal stem cell exosomal miRNAs promote lung cancer cell metastasis through exosome-mediated transfer of miRNAs by activating the signal transducer and activator of transcription 3 signaling-induced epithelial-mesenchymal transition [14].

Although many researchers have found that various EV-derived YRNAs and YRNA fragments are related to lung cancer, their functions and the underlying mechanisms involved in promoting tumor progression are inconclusive. Previously, using high-throughput sequencing of small RNAs in plasma EVs from non-small cell lung cancer (NSCLC) patients and healthy controls, we found that a variety of small non-coding RNA species are present in plasma EVs. Our results demonstrate that hY4-derived fragments (hY4F) are significantly upregulated in plasma EVs from NSCLC patients and may be promising biomarkers for NSCLC [15]. Additionally, we found that hY4Fs were significantly downregulated in NSCLC cells, suggesting that the selective sorting of hY4F into EVs plays an important role in NSCLC progression. Based on these results, we sought to investigate why hY4F is highly enriched in lung cancer-derived EVs, whether EV hY4F plays a role in lung cancer progression, and further to determine the mechanism of hY4F sorting into EVs.

In this study, we reveal that hY4F are upregulated in NSCLC-derived EVs, and that the selective sorting and secretion of hY4F into lung cancer EVs is regulated by the RNA-binding protein Y box binding protein 1 (YBX1). Furthermore, we found that hY4F is a tumor suppressor and that its secretion into EVs is involved in the proliferation, migration, and invasion of lung cancer cells. We identified lysine K264 within the YBX1 C-terminal domain as necessary for its interaction with hY4F and subsequent selective sorting of hY4F into EVs. Finally, our results demonstrate that K264 may be modified by methylation, which affects its binding to hY4F and selective sorting into EVs in lung cancer cells.

## Materials and methods

### Cell culture

Human embryonic lung fibroblasts (IMR-90) and human lung adenocarcinoma cell lines (A549, H1975, H460, and H2030) were purchased from the Cell Bank of the Chinese Academy of Sciences (Shanghai, China). IMR-90 was cultured in MEM medium (Gibco, USA), while A549, H460, and H2030 were cultured in DMEM medium (Gibco, USA). H1975 cells were cultured in RPMI 1640 medium (Gibco, USA). All cells were cultured with medium supplemented with 10% fetal bovine serum (FBS) (Gibco, USA), 100 U/ml penicillin (Biosharp, China), and 100 U/ml streptomycin (Biosharp, China) at 37 °C with 5% CO_2_.

### EVs isolation

EVs were purified from cells-conditioned media by differential ultra-centrifugation [16]. FBS (VivaCell, China) used for EVs isolation was depleted of EVs by ultracentrifugation at 100,000 x g overnight at 4 °C. Then the supernatant was sterilized through a 0.45 μm filter (Millipore, USA) and stored at − 20 °C. When the cell culture density reached 50%, the medium was replaced with EV-depleted medium. Conditioned media were collected after 48 h and centrifuged at 300 x g for 10 min, 2000 x g for 20 min, and 10,000 x g for 40 min at 4 °C to remove cells and debris. The EVs were harvested by centrifugation at 100,000 x g for 90 min (XE-100, Beckman Coulter, USA). The pellets were then washed with phosphate-buffered saline (PBS) to remove any contaminating protein and then resuspended in PBS. Quantification of EVs was performed using the BCA assay (Pierce, USA) with surface proteins.

### Oligonucleotide and transfection

A549 and H1975 were transfected with hY4F RNA mimics (GGCUGGUCCGAUGGUAGUG-GGUUAUCAGAACU) or NC at a final concentration of 50 nM. And siRNA targeting hY4F (sense: UUAAUAAGUUCUGAUAACCTT, anti-sense: GGUUAUCAGAACUUAUUAATT) was transfected into IMR-90 cells at a final concentration of 150 nM. Other siRNAs used in this paper were transfected into cells at a final concentration of 50 nM. RNA oligos used in this paper, including siRNA and RNA mimic, were purchased from Genepharma (Suzhou, China). Both RNA mimic and siRNA in this paper were transfected using Lipofectamine® 2000 reagent (Invitrogen), following the manufacturer’s instructions.

### Western blot and immunoprecipitation analysis

The cell pellets were separately re-suspended in RIPA buffer (Biosharp, China) supplemented with protease inhibitors (Roche, Switzerland). Total protein (20 μg) was then separated on a 10% SDS-PAGE gel (Thermo Fisher Scientific, USA). Proteins were transferred to a polyvinylidene fluoride (PVDF) membrane (Millipore, USA), and blocked with 5% skim milk (BD, USA) in Tween/Tris buffered saline (TTBS) for 1 h at room temperature. Membranes were then incubated with primary antibodies against YBX1 (1:3000, Proteintech), Flag tag (1:10,000, Abcam), SETD3 (1:500, ABclonal) GAPDH (1:20,000, Proteintech), HSP90 (1:20,000, Abcam), Ro60 (1:3000, Proteintech), RPS26 (1:3000, Proteintech), hnRNPA2B1 (1:3000, Proteintech), HNRNPH1 (1:3000, Proteintech), or pan di-methyl-lysine (1:1000, A18296, ABclonal) with 3% skim milk or bovine serum albumin diluted in TTBS for 1 h at room temperature. After washing several times with TTBS, membranes were incubated with horseradish peroxidase (HRP)-conjugated IgG secondary antibodies (Santa Cruz Biotechnology, USA) at a dilution of 1:10,000 in 3% skim milk diluted in TTBS for 1 h at room temperature. After washing several times with TTBS, enhanced chemiluminescence was performed with ECL substrate (Bio-Rad, USA) and bands were visualized by exposing the membrane to film and developing in a film processor.

For immunoprecipitation, equal amounts of the cell lysates were incubated with Dynabeads Protein G (Thermo Fisher Scientific) conjugated with specific antibody at 4 °C overnight. Next, the precipitants were washed four times with lysis buffer. And the immunocomplexes were eluted with sample buffer containing SDS loading buffer for 10 min at 95 °C, then separated by SDS-PAGE.

### Quantitative real-time PCR (qPCR) analysis

Total RNA was isolated from cells and EVs using Trizol reagent (Invitrogen, Life Technologies, USA). The concentration and quality were assessed using the Nanodrop2000 (Thermo Fisher Scientific). RNA was reverse transcribed using the HifairTM II 1st Strand cDNA Synthesis Kit (Yeasen, China). Quantitative real-time PCR analysis was performed on the ABI7500 real-time PCR amplifier (Applied Biosystems, USA) using SYBR Green Master Mix (Yeasen, China). U6 or cel-miR-39 was used as a control, and results were analyzed using the 2^–ΔΔct^ method.

### EdU incorporation assay

EdU (KeyGEN BioTECH, China) was added to the cell culture medium to a final concentration of 20 μM before incubation at 37 °C for 4 h. The cells were then fixed with 4% paraformaldehyde (Biosharp, USA) for 20 min at room temperature, washed with PBS, and treated with 0.5% Triton X-100 (Sigma, Germany) for 20 min at room temperature. Next, the reaction buffer and KFlour488-azide (KeyGEN BioTECH, China) were mixed and incubated with the cells at room temperature protected from light for 30 min. Lastly, the cells were washed with PBS twice and incubated with Hoechst33342 solution (KeyGEN BioTECH, China) at room temperature in the dark for 30 min.

### Cell migration and invasion assay

Cell migration was examined using transwell chambers (0.8 μm 24-well plates, Corning, USA). Cells (1 × 10^4^) were suspended in serum-free medium (200 μl) and seeded into the chambers before 500 μl of medium containing 10% FBS was added to the bottom. After a 24 h incubation, cells were fixed with 4% paraformaldehyde and stained with 1% crystal violet solution (Biosharp, USA). Then the cells were observed by microscopy by randomly choosing six fields to count the number of migrated cells. For cell invasion analysis, matrix gel (Corning) was added to the transwell chambers before cell seeding; all other procedures were the same.

### Cell proliferation assay

Cells were seeded in a 96-well plate (5 × 10^3^ cells/well) and cultured for 24, 48, and 72 h. Cell Counting Kit 8 (CCK-8) reagent (TargetMol, China) mixed with DMEM medium (10 μl: 90 μl) was added to each well and incubated for 1 h. Then, the absorbance at 450 nm was measured using a microplate reader (Bio-Rad, USA). The absorbance of each well was measured at least three times, and the mean absorbance was used to assess cell proliferation.

### Colony formation assay

After transfected for 24 h, cells are seeded into 6-well culture plates in dilutions of 200 cells per well to form colonies in 7 days. Colonies are fixed with 4% (v/v) glutaraldehyde for 15 min, stained with 1% (w/v) crystal violet for 20 min, and counted using a stereomicroscope.

### Assessing apoptosis by flow cytometric analysis

Adherent cells (5 × 10^5^) were collected with trypsin digestion without EDTA and then washed with PBS twice (centrifugation at 2000 rpm for 5 min). Then, 500 μl binding buffer was added to the resuspended cells followed by 5 μl Annexin V-FITC and mixed well. Next, 5 μl propidium iodide (PI) was added and the samples were mixed at room temperature away from light for 5–15 min. After resuspension and filtration, the samples were analyzed by flow cytometry (CytoFLEX, Beckman, USA) within 1 h.

### Flow cytometric cell cycle analysis

Analysis of cell cycle distribution was carried out by flow cytometric analysis of propidium iodide (PI)-stained cells. The cells (1 × 10^6^) were fixed with 75% ethanol for 4 h at 4 °C. The samples were then centrifuged at 300 g for 5 min. Then 75% ethanol was removed, and the cells were treated with 20 mg/mL of PI containing RNase A (0.5 mg/ml) for 30 min at RT. And cell cycles were analysed with a Beckman Cytofelx flow cytometer to obtain DNA content profiles. FlowJo was used for the analysis of cell cycle distribution.

### Biotin-labelled RNA pulldown

The Thermo Fisher Scientific Pierce RNA 3′ End Desthiobiotinylation Kit was used to attach a single desthiobiotinylated cytidine bisphosphate to the 3′-end of hY4F or the control RNA. Then, the biotinylated RNA was incubated with A549 cell lysates to enrich for proteins that bind to hY4F. Western blotting and liquid chromatography-tandem mass spectrometry (LC-MS/MS) were used to analyze the RNA binding proteins isolated by streptavidin beads.

### Biotin-labelled peptide pulldown

The biotinylated mono/di/tri-methyl-lysine (YBX1-K264) peptide and unmodified control peptide (QPREDGNEEDKENQGDETQGQQ) were synthesized (GL BioChem, China). Then the peptides were incubated with A549 cell lysates to enrich for proteins that bind to them. LC-MS/MS was used to analyze the potential methyltransferase which binds to methyl-lysine peptides.

### Biotin-labelled peptide dot blot

The above biotin-labelled peptides with unmodified or mono/di/tri-methyl-modified lysine (K264) of YBX1 were used for the identification of methyl-lysine antibodies. The nitrocellulose membrane was labeled using a pencil, and 2 μl from each fraction were pipetted onto the membrane, allow solution adsorbed by the membrane. After dried, membrane was incubated in blocking solution for 1 h, and then treated with primary antibody solution (diluted in blocking solution) for 2 h at room temperature. Next, membrane was washed with washing buffer (3 × 10 min), and then incubated with HRP-conjugated secondary antibody (in blocking solution) for 1 h. After washing 3 × 10 min with washing buffer, enhanced chemiluminescence was performed with ECL substrate (Bio-Rad, USA) and dots were visualized by exposing the membrane to film and developing in a film processor.

### Liquid chromatography-tandem mass spectrometry (LC-MS/MS)

The LC-MS/MS analysis was performed on an EASY-nLC™ 1000 (Thermo Fisher Scientific) coupled to a Q Exactive™ HF (Thermo Fisher Scientific). The loading of the samples was performed on Nano Trap Column (Thermo Fisher Scientific). The compositions of the mobile phase A and B were 0.1% formic acid (A), 0.1% formic acid and 0.1% acetonitrile (B); 5 μl of the sample was injected. The sample was taken into the analytical column for separation through a 60-min chromatographic gradient. The column was equilibrated for 5 min and the column temperature was set to 35 °C. After flowing out of the chromatographic column, samples passed through the Nano Flex Ion Source under the spray voltage of 2.4 kV. Then the charged sample is sprayed into the mass spectrometer for detection, and the mass range was set from m/z 300 to 1800. Alignment of the spectra, peak picking and further analysis were processed using the Proteome Discoverer software (Thermo Fisher Scientific).

### Transcriptome sequencing

Sequencing libraries were generated as follows: following isolation of total RNA from triplicate samples of NC/hY4F mimic transfected A549 cells, RNA samples were analyzed with an Agilent2100 Bioanalyzer (Agilent) using a total RNA nanochip. Next, the cDNA library was prepared using the TruSeq Stranded mRNA Prep Kit (Illumina), and was sequenced using the HiSeq2500 platform (Illumina). To get clean reads, the adaptor sequences, contaminated reads, low quality reads, less than 17 nt reads, and poly-A/T/C/G/N repeat sequences were removed. Next, the clean reads were mapped to the human genome database using Burrows-Wheeler Aligner software. In addition, counts per feature were normalized using the reads per kilobase of transcript per megabase library size (RPKM) method. Significant differences in mRNA expression were determined using R package edgeR, with thresholds of a *P*-value < 0.05, a false discovery rate (FDR) < 0.05, and a |log2 (Fold Change)| ≥0.58. The heatmap of mRNA expression differences was plotted using function heatmap.2 in R package gplots. The GO enrichment analysis of target genes was performed with the BinGO plugin for Cytoscape software version 3.4.0 using whole annotation as a reference set; hypergeometric test, false discovery rate (FDR) correction, *P* < 0.05 significance level and GO BP (Biological Process) ontology files were selected. For KEGG pathway enrichment analysis, the DAVID online tool (https://david.ncifcrf.gov) [17] was used with Fisher’s exact test, FDR c orrection, and *P* < 0.05 significance level. 

### Animal experiments

All animal experiments were performed in accordance with a protocol approved by the Institutional Animal Care and Use Committee of Wuhan University. To analyze tumorigenesis, 5-weeks-old male BALB/c-nude mice were injected subcutaneously with 1 × 10^7^ A549 cells per mouse. The tumor volume of nude mice was measured twice a week, and the formula v = 1/2 x a x b^2^ (v means volume, a means long diameter, and b means short diameter) was used to calculate the tumor volume. When the experiments were completed, the mice were sacrificed and the tumors were dissected and weighed. To detection tumor metastasis ability, BALB/c-nude mice were injected via tail vein with 2 × 10^6^ A549 cells per mouse, and lungs metastasis of mice were analyzed by H&E stain method 5 weeks later.

### Clinical samples

Clinical plasma samples were collected in the Wuhan Tongji Hospital and Hubei Cancer Hospital with written informed consent from all human participants. And tumor tissues and paired plasma of lung adenocarcinoma patients were collected in BioBank of Hubei Cancer Hospital with all individuals’ written informed consent. Approval for the study was granted by the Institute Research Ethics Committee at Wuhan University.

### Statistical analysis

All values are presented as mean ± standard deviation (SD). Significant differences were determined using GraphPad 5 software. The Student’s t-test was used to determine statistical differences between the two groups. Survival curves were plotted using the Kaplan-Meier method and compared by log-rank test. *P* < 0.05 was considered statistically significant. Experiments were repeated three times where possible.

## Results

### hY4 RNA fragments are selectively sorted into lung cancer cell-derived EVs

**Fig. 1:**
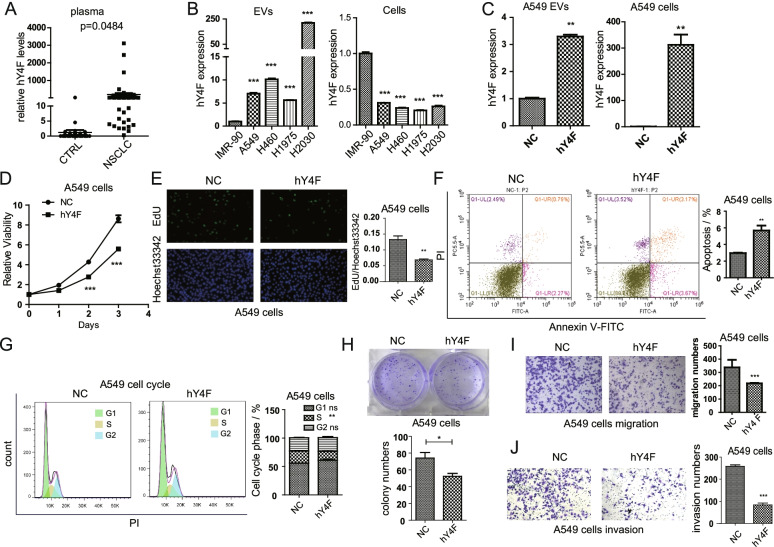
hY4 RNA fragments are selectively sorted into lung cancer cell-derived EVs and inhibit cancer cell progression. **A** The level of hY4F RNA in plasma from healthy control subjects (CTRL, *n* = 34) and non-small cell lung cancer patients (NSCLC, *n* = 49) was assessed by qPCR. **B** The level of hY4F in EVs and cells from normal lung fibroblast (IMR-90) and lung cancer cells (A549, H460, H1975 and H2030) was examined by qPCR. **C** The hY4F level in EVs and cell lysates from hY4F mimic/NC-transfected A549 cells was measured by qPCR (at 48 h after transfection). The effect of a hY4F mimic on A549 cell proliferation was examined by (**D**) CCK-8 (at 24, 48, and 72 h after transfection) and (**E**) EdU assays (at 48 h after transfection). **F** Flow cytometry using Annexin V-FITC/PI staining was performed to examine apoptosis of hY4F-overexpressing A549 cells (at 24 h after transfection). **G** Cell cycle analysis by PI staining-based flow cytometry with hY4F mimic/NC-transfected A549 cells (at 24 h after transfection). **H** Colony formation assay with A549 cells at 7 d after transfected with hY4F mimic/NC. The effect of a hY4F mimic on A549 (I) migration and (**J**) invasion was assessed by transwell assay (at 24 h after transfection). Data from three independent experiments are shown as the mean ± SD (error bars). *P < 0.05, **P < 0.01, ***P < 0.001 (Student’s t-test). NC: negative control

Although many studies have demonstrated that YRNA-derived fragments in EVs can serve as potential biomarkers for various cancers [18, 19], the biological function and molecular mechanisms of YRNA-derived fragments remain unclear. To investigate hY4F expression in the circulation of NSCLC patients, we measured hY4F levels in plasma. The qPCR results showed that hY4F is dramatically enriched in the plasma of NSCLC patients (Fig. 1A and Table. S1) similar to EVs [15]. And both lung adenocarcinoma (LUAD) and lung squamous cell carcinoma (LUSC) patients showed up-regulated expression of plasma hY4F compared to healthy controls. Nevertheless, there was no significant difference of plasma hY4F levels between patients with different histology or stage (Fig. S1A). We also assessed the expression of hY4 RNA fragments, such as hY4F, in EVs derived from NSCLC cell lines (A549, H460, H1975, H2030) compared to the normal lung fibroblast cell line IMR-90. The results show that hY4F was also significantly upregulated in NSCLC cell lines-derived EVs but downregulated in NSCLC cells (Fig. 1B).

Next, we tested whether hY4F affects the proliferation, migration, and invasion of NSCLC cells. We transfected hY4F mimic or negative control in A549 cells, and our data demonstrate that overexpression of hY4F in A549 cells results in a significantly increased level of hY4F in its EVs (Fig. 1C). Similarly, transfection of hY4F mimic in H1975 cells increased level of hY4F in H1975 EVs (Fig. S1B). Results from CCK-8 and EdU assays demonstrated that overexpression of an hY4F mimic significantly suppressed the proliferation of A549 (Fig. 1D and E) and H1975 (Fig. S1C and D) lung cancer cells. Flow cytometric analysis revealed that the percentage of apoptotic cells was significantly increased in hY4F-overexpressing lung cancer cells (Fig. 1F and S1E). In addition, we analyzed the influences of hY4F overexpression on cell cycle and colony formation. Results of cell cycle assay showed that transfection of hY4F significantly inhibited the ratio of S phase and G1/S phase transition (Fig. 1G). However, the influences of cell cycle were not obvious as apoptosis. And colony formation assay indicated that hY4F suppressed clonogenic ability of lung cancer cells (Fig. 1H). Transwell assays showed that hY4F inhibited the migration and invasion of A549 (Fig. 1I and J) and H1975 (Fig. S1F and G) cells. These data suggest that hY4F functions as a tumor suppressor that inhibits NSCLC proliferation, migration, and invasion, possibly by regulating lung cancer cell apoptosis and cell cycle.

Considering that hY4F is overexpressed in normal lung cells compared to cancer cells, we analyzed the effects of hY4F silencing in IMR-90 cells. SiRNA target hY4F was transfected into A549 cells, and the expression of hY4F was significantly suppressed by approximately 80% (Fig. S2A). And knockdown of hY4F obviously increased the proliferation of IMR-90 cells (Fig. S2B). The above results suggested that hY4F inhibited cell proliferation in both normal and cancer cells.

Furtherly, we investigated whether the selective sorting of hY4F into EVs plays a role in lung cancer progression. When EVs from hY4F-overexpressing A549 cells were incubated with H1975 cells, the hY4F level was increased significantly in H1975 cells (Fig. S3A). Treatment of H1975 cells with hY4F-overexpressed A549 EVs obviously inhibited cell proliferation (Fig. S3B). And flow cytometric analysis indicated that the percentage of apoptotic cells was significantly increased in hY4F-EVs treated lung cancer cells (Fig. S3C). Moreover, transwell experiments showed that incubation of H1975 cells with A549 EVs combined with hY4F overexpression suppressed H1975 cell migration and invasion (Fig. S3D and E). Taken together, these results indicate that selective sorting of hY4F into EVs inhibits the proliferation, migration, and invasion of lung cancer cells.

### hY4 RNA fragments inhibit proliferation and migration of lung cancer cells through downregulating MAPK/NF-κB signaling

**Fig. 2:**
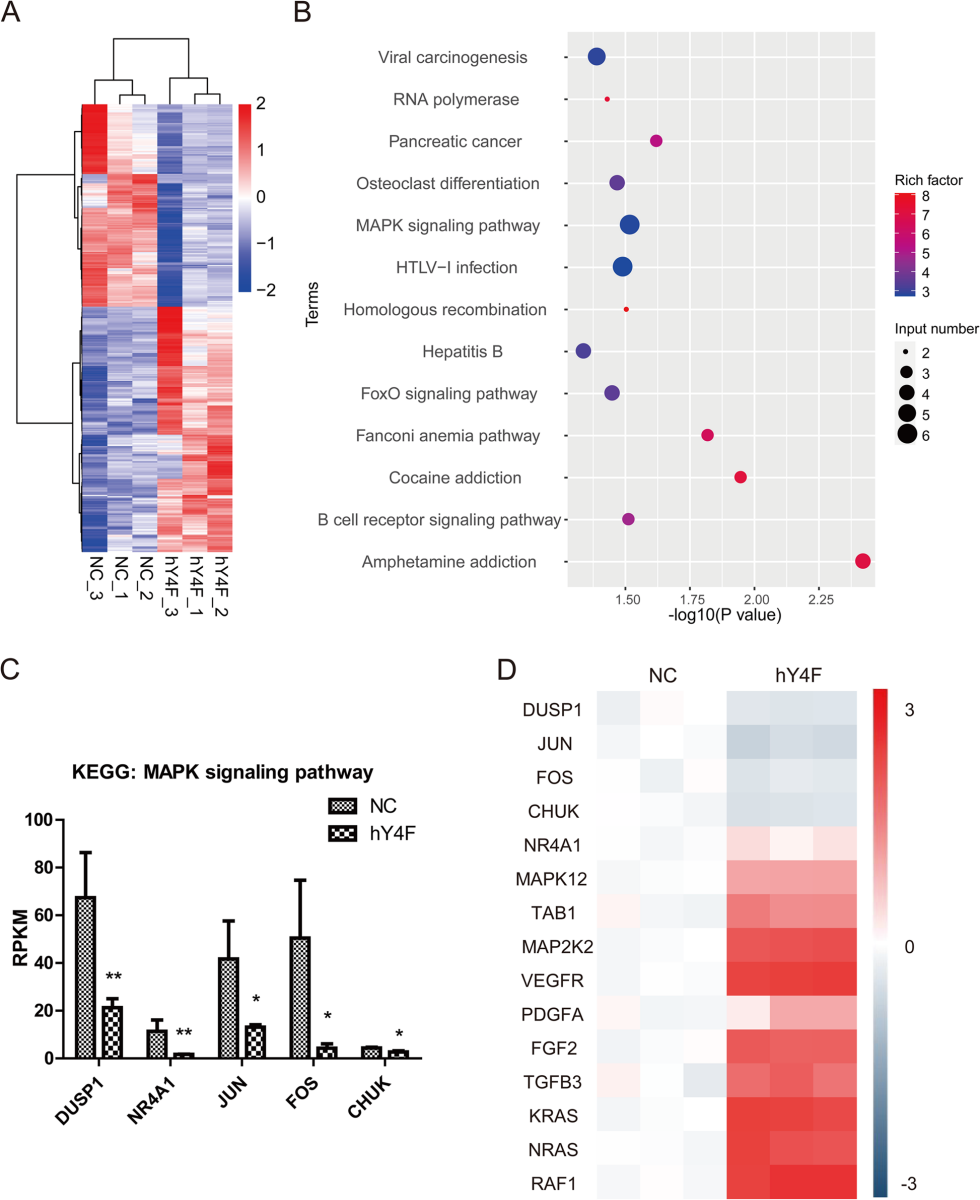
hY4F inhibits lung cancer progression through MAPK/NF-κB pathway. **A** The results of differentially expressed genes (DEG) cluster analysis presented by heatmap in A549 cells transfected with hY4F mimic/NC for 24 h. **B** KEGG pathway enrichment analysis of genes in response to hY4F overexpression. **C** Transcriptional sequencing results of five coding genes enriched into MAPK pathway. **D** QRT-PCR analysis results of the MAPK pathway related genes in Fig. 2A

To elucidate the mechanism of hY4F inhibiting lung cancer, we performed transcriptome sequencing using lung cancer cells transfected with hY4F mimic/NC. To determine whether genes are differentially expressed between NC group and hY4F group, clean sequencing reads were mapped to reference genome (hg19) and normalized by Reads per Kilobase per Million Reads (RPKM) method. The analysis results of differentially expressed genes (DEG) showed that there are 211 genes downregulated and 174 genes upregulated in hY4F overexpressed A549 cells (Table S2). The results of gene cluster analysis are presented by heatmap (Fig. 2A). And the KEGG pathway enrichment analysis (Fig. 2B) showed that significantly downregulated genes were enriched into MAPK signaling pathway in response to hY4F overexpression. Among these enriched genes, five coding genes DUSP1, NA4A1, JUN, FOS, and CHUK were MAPK pathway genes (Fig. S4) and downregulated by hY4F (Fig. 2C). In addition, the expression of the above DEGs, as well as other genes playing important roles in MAPK pathway, were analyzed by qRT-PCR analysis (Fig. 2D). And the above qRT-PCR results further confirmed that four genes DUSP1, JUN, FOS, and CHUK (IKKα) were downregulated by hY4F. Among these genes, CHUK is the upstream kinase of NF-κB, and can activate NF-κB signaling. The above results suggested that hY4F may inhibit lung cancer progression through downregulating MAPK/NF-κB pathway.

However, several oncogenes were showed to be upregulated in cells treated with hYF4 mimic in the above results. It could be speculated that the effects of NSCLC-derived EVs containing hY4F could have pro-oncogenic activities on normal cells. Thus, IMR-90 cells were exposed to hYF4-A4549 EVs to analyze proliferation and expression of related genes. The results showed that treatment of hY4F-overexpressed A549 EVs increased hY4F level (Fig. S5A) and promoted proliferation (Fig. S5B) of IMR-90 cells. And qRT-PCR results indicated that several oncogenes, such as NRAS, RAF1 and VEGFR, were obviously up-regulated (Fig. S5C).

### YBX1 binds hY4F to regulate its selective sorting into lung cancer EVs

**Fig. 3:**
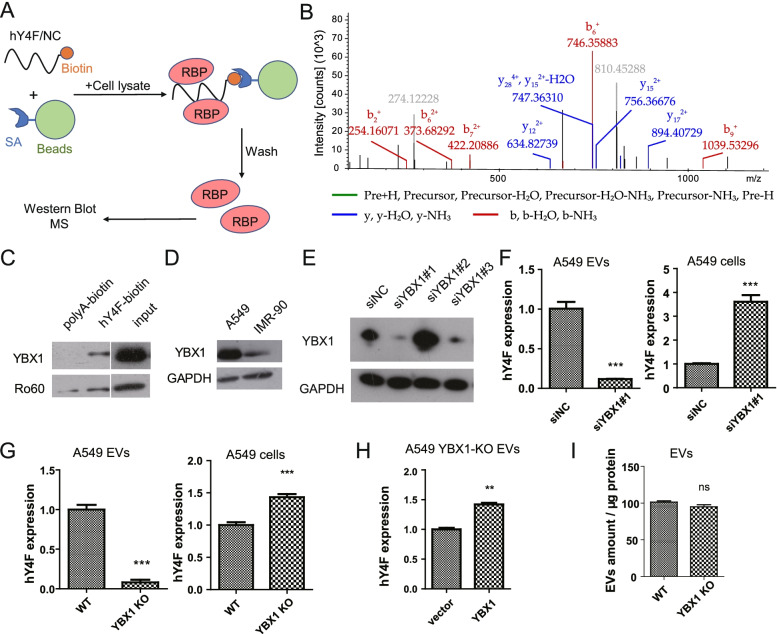
YBX1 protein binds hY4F to regulate its sorting into lung cancer EVs. **A** Schematic representation of the biotin-labelled hY4F RNA pulldown assay. **B** Representative MS/MS data of specific peptides of YBX1 identified in this paper. **C**) Western blot analysis of hY4F-binding proteins enriched by the RNA pulldown assay. **D** Western blot analysis of YBX1 and Ro60 in IMR-90 and A549 cells. **E** Western blot analysis of siRNA-mediated YBX1 knockdown of A549 cells after transfection for 24 h. And #1, #2, and #3 means different siRNAs targeting YBX1. **F** Quantitative PCR results of hY4F in EVs and cell lysates from A549 transfected with siRNA (#1) targeting YBX1 for 48 h. **G** Quantitative PCR results of hY4F in EVs and cell lysates from wild type and YBX1 knockout (KO) A549 cells. **H** Quantitative PCR data of hY4F in EVs from A549-YBX1 KO cells overexpressing YBX1 or an empty vector. **I** Quantitative data of wild type and YBX1-KO A549 EVs using the surface protein BCA assay. Data from three independent experiments are shown as the mean ± SD (error bars). *P < 0.05, **P < 0.01, ***P < 0.001. ns: no significant difference (Student’s t-test). NC: negative control

Studies have shown that some ncRNAs, including hY4 RNA, are selectively released into EVs, and that RNA-binding proteins are necessary for this selective sorting [20–22]. Based on this research, we sought to identify the RNA-binding protein that interacts with hY4F. We performed RNA pulldown experiments whereby synthetic biotin-labeled hY4F was incubated with a cell lysate, and then the proteins bound to hY4F were eluted and verified by mass spectrometry and western blot (Fig. 3A). Mass spectrometry identified several proteins that could potentially interact with hY4F (Fig. 3B and Table S3), and some of them, including leucine-rich PPR-motif containing protein (LRPPRC), YBX1, heterogeneous nuclear ribonucleoproteins A2/B1 (hnRNPA2B1), hnRNPH1, ribosomal protein S26 (RPS26), and Ro60, were validated by western blot (Fig. 3C and S6A). One candidate, YBX1, is an RNA-binding protein involved in the sorting of a variety of small RNAs [20, 21]. We confirmed the interaction of hY4F with YBX1 by western blot using a specific antibody against YBX1. Ro60 is a known protein that binds to YRNA to form RoRNP and was used as a positive control (Fig. 3C). Moreover, we found that the level of YBX1 was higher in A549 cells than in IMR-90 cells (Fig. 3D).

Next, we examined the effect of siRNA-mediated YBX1 depletion in A549 cells. Our data show that the level of hY4F was significantly reduced in A549 EVs after YBX1 knockdown even though the content of intracellular hY4F was increased (Fig. 3E and F). We also confirmed that YBX1 knockdown in H1975 cells also decreased the hY4F sorting into EVs and subsequent secretion (Fig. S6B), suggesting that YBX1 may mediate the selective sorting of hY4F into EVs.

Then, we used CRISPR/Cas9 methods to knock out *YBX1* in A549 cells. These experiments revealed that the level of hY4F sorted into EVs was significantly reduced in the absence of YBX1 protein (Fig. 3G and S6C), indicating that YBX1 is necessary for the sorting and secretion of hY4F into lung cancer EVs. Overexpressing YBX1 into YBX1-KO A549 cells restored the content of hY4F-sorted EVs (Fig. 3H). And YBX1 knockout did not influence the mounts and sizes of EVs secreted by A549 cells (Fig. 3I and S6D). In addition, the effect of YBX1 knockout was specific to hY4F as the other three human YRNAs did not exhibit any increased sorting into EVs (Fig. S6E and F). Collectively, these results indicate that YBX1 binds hY4F to regulate its sorting and secretion into EVs in lung cancer.

### YBX1 is involved in lung cancer cell proliferation and migration

**Fig. 4:**
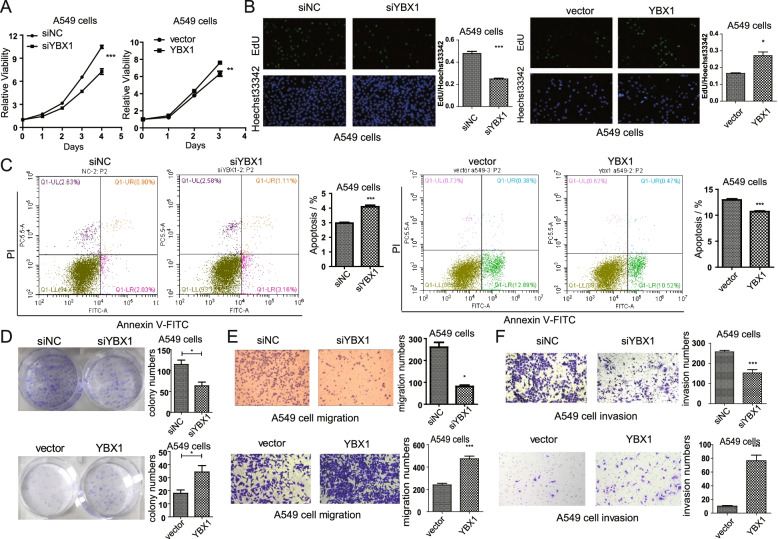
YBX1 promotes lung cancer cell proliferation and migration. **A** CCK-8 (at 24, 48, 72 and 96 h after transfection) and (**B**) EdU assays (at 48 h after transfection) were performed to assess the effect of YBX1 knockdown and over-expressing on A549 cell proliferation. **C** Flow cytometry using Annexin V-FITC/PI staining was performed to analysis apoptosis of YBX1 knockdown and overexpressing A549 cells (at 24 h after transfection). **D** Colony formation assay with A549 cells at 7 d after knockdown and overexpression of YBX1.The effect of YBX1 knockdown and overexpression on A549 (**E**) migration and (**F**) invasion was examined by transwell assay (at 24 h after transfection). Data from three independent experiments are shown as the mean ± SD (error bars). *P < 0.05, **P < 0.01, ***P < 0.001 (Student’s t-test). NC: negative control

Next, we studied whether YBX1 plays a vital role in lung cancer progression. Analysis of cell viability using CCK-8 assay (Fig. 4A) and EdU assay (Fig. 4B) showed that knockdown of YBX1 inhibited lung cancer cell proliferation, while overexpression of YBX1 promoted proliferation. Flow cytometry results showed that the percentage of apoptotic cells was significantly increased in YBX1 downregulated lung cancer cells, while decreased in YBX1 overexpressed cancer cells (Fig. 4C). And colony formation assay indicated that knockdown of YBX1 inhibited the clonogeinc ability of A549 cells, while overexpression of YBX1 promoted colony formation (Fig. 4D). In addition, transwell assays demonstrated that YBX1 knockdown suppresses lung cancer cell migration and invasion, while overexpression of YBX1 promoted cell migration and invasion (Fig. 4E, F). These results were also observed in H1975 cells (Fig. S7A-E), suggesting that YBX1 may regulate cell proliferation, migration, and other processes by affecting cell apoptosis.

**Fig. 5:**
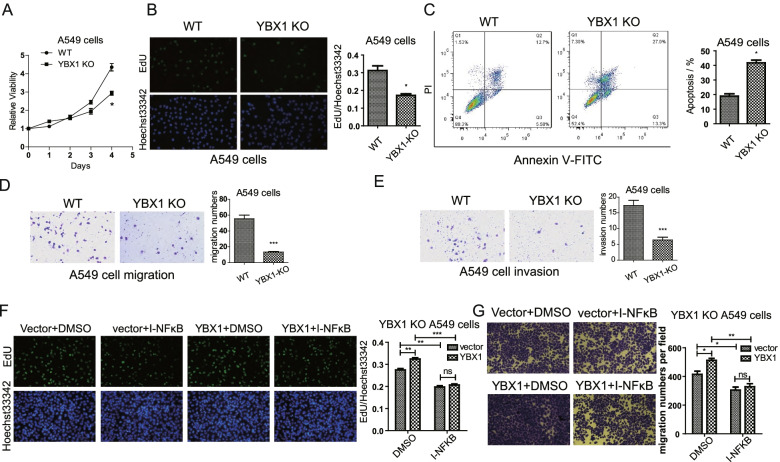
YBX1 regulates lung cancer cell progression through NF-κB pathway. Results from (**A**) CCK-8 (at 24, 48, 72 and 96 h after transfection) and (**B**) EdU assays (at 48 h after transfection) showing the effect of YBX1 knockout (KO) on A549 cell proliferation. (**C**) Flow cytometry using Annexin V-FITC/PI staining was performed to analyze apoptosis of YBX1 knockout A549 cells (at 24 h after transfection). The effect of YBX1 knockout on A549 (**D**) migration and (**E**) invasion of A549 cells was examined by transwell assay (at 24 h after transfection). **F** EdU assays showing the influences of YBX1 plasmid transfection and NF-κB inhibitor (IMD-0354, 5 μM, 24 h) treatment on cell proliferation in YBX1-KO A549 cell (at 48 h after transfection). **G** Transwell assays for analysis the influences of YBX1 plasmid transfection and NF-κB inhibitor (IMD-0354, 5 μM, 24 h) treatment on cell migration in YBX1-KO A549 cell (at 36 h after transfection). Data from three independent experiments are shown as the mean ± SD (error bars). *P < 0.05, **P < 0.01, ***P < 0.001 (Student’s t-test). NC: negative control; WT: wild type

In addition, we found that, YBX1-knockout A549 cells showed lower proliferation compared with wild-type A549 cells (Fig. 5A, B). And flow cytometry analysis demonstrated that the apoptotic ratio was significantly increased in YBX1 knockout lung cancer cells (Fig. 5C). Besides, transwell assays indicated that knockout of YBX1 inhibited migration (Fig. 5D) and invasion ability (Fig. 5E) in A549 cells.

Considering that YBX1 regulates hY4F secretion and hY4F downregulates MAPK/NF-κB related genes, we wonder whether YBX1 also influences NF-κB pathway. To test the function of NF-κB signaling pathway in YBX1 induced tumor progression, we investigated the effect of a specific inhibitor of NF-κB pathway, IMD-0354. The YBX1 knockout A549 cells were treated with IMD-0354/DMSO and transfected with YBX1 plasmid/empty vector. The EdU (Fig. 5F) and transwell assay (Fig. 5G) showed that the exogenous YBX1 regained the proliferation and migration ability of A549 cells, and inhibiting NF-κB signaling with IMD-0354 resulted in drastically reduced YBX1 induced tumor progression. These results indicate that YBX1 promotes proliferation, migration and invasion of lung cancer cells, and YBX1 induces tumor progression through EVs-hY4F sorting associated MAPK/NF-κB signaling axis.

### Methylation of YBX1 K264 regulates hY4F sorting into lung cancer EVs

**Fig. 6:**
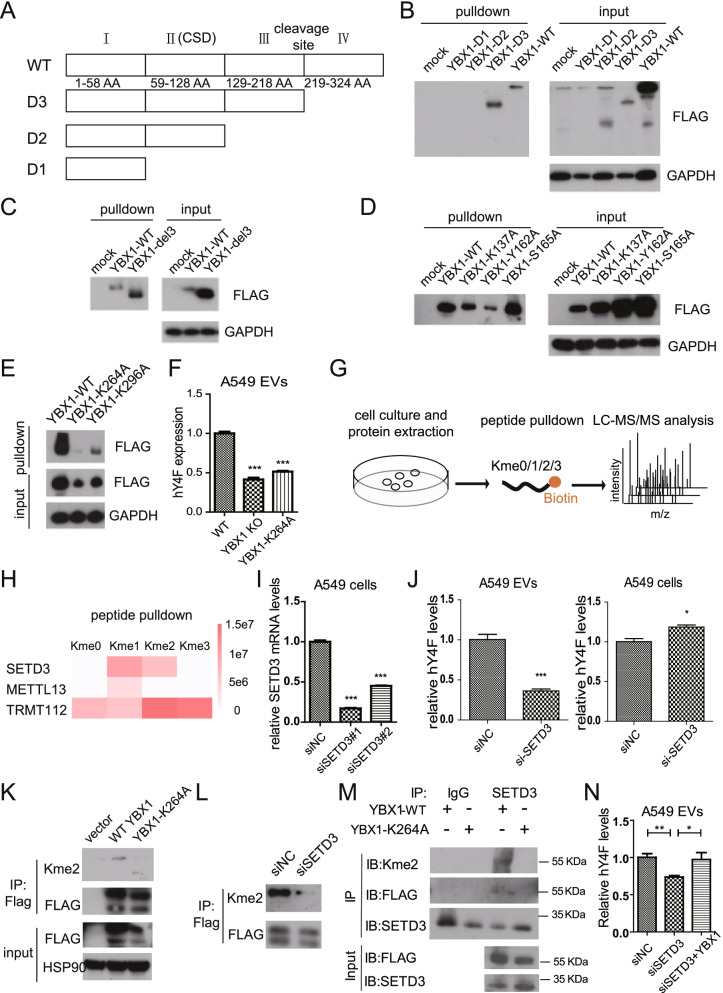
SETD3 regulates the sorting of hY4F into EVs through K264 methylation on YBX1. **A** Schematic representation of the functional domains of YBX1. **B**-**E** Biotin-labelled hY4F RNA pulldown analysis with cell lysate from A549 cells overexpressing FLAG-tagged truncated or mutated YBX1. **F** Quantitative PCR data of hY4F in EVs from YBX1-KO A549 cells transfected with wild type (WT) or K264A mutant YBX1. **G** Schematic representation of the pulldown assay using biotin-labelled unmodified or mono/di/tri-methyl-lysine modified YBX1-K264 peptide. **H** The quantitative mass spectrometry results of peptide pulldown assay shown as heatmap. **I** Verification of siRNA-mediated knockdown of SETD3 in A549 cells by qPCR. **J** The effect of siRNA-mediated SETD3 knockdown on hY4F level in EVs and cell lysates from A549 cells transfected with SETD3 siRNA. **K** Methylation of lysine K264 identified by di-methyl-lysine (Kme2) antibody. **L** The effect of SETD3 knockdown on YBX1 methylation level was assessed by western blot. **M** Analysis of YBX1 methylation by SETD3 in vitro using endogenous immunoprecipitation assay using SETD3 antibody in the presence of wild-type or K264A mutant. **N** QPCR analysis of EVs-hY4F levels in A549 co-transfected with YBX1 expression plasmid and siRNA targeting SETD3. Data from three independent experiments are shown as the mean ± SD (error bars). *P < 0.05, **P < 0.01, ***P < 0.001 (Student’s t-test)

Since RNA-binding proteins typically interact with RNA through specific domains, we aimed to identify the YBX1 domains that interact with hY4F. The YBX1 protein is divided into three parts (Fig. 6A): the N-terminal AP domain (domain I), the CSD structure (domain II), and the C-terminal domain, which can be further divided into two domains (domains III and IV) based on the cleavage site. We constructed a series of truncated eukaryotic YBX1 overexpression plasmids, transfected them into A549 cells, and then performed pulldown assays. Our results demonstrate that the C-terminal domains III and IV of YBX1 is necessary for its interaction with hY4F (Fig. 6B, C).

Studies have shown that post-translational modifications of RNA-binding proteins play important roles in regulating RNA sorting into EVs. For example, SUMOylation modification of hnRNPA2B1 is necessary for the selective sorting of miRNAs into EVs [23]. Therefore, we proceeded to screen for and identify the key amino acids within the C-terminal domain of YBX1 that interact with hY4F. We constructed plasmids that express YBX1 mutants in which potential acetylation, phosphorylation, or methylation sites within the C-terminal domain are mutated to alanine. These constructs were transfected into A549 cells, and then cell lysates were co-incubated with biotin-labeled hY4F RNA before pulldown assays were performed. Our results show that K137 and Y162 on domain III and K264 on domain IV of YBX1 play a role in hY4F binding (Fig. 6D, E). Furthermore, we simulated methylation of YBX1 K137 and K264 by mutating the lysines to methionine and found that the above mutations were still supportive for YBX1 binding to hY4F (Fig. S8A). These results suggested that potential methylation of YBX1 may promote its binding to hY4F. In addition, we simulated the acetylation and non-acetylation states of YBX1 by mutating these lysines to glutamine or arginine, respectively. The RNA pulldown results suggest that potential acetylation of K137 and K264 was not required for hY4F binding (Fig. S8B).

Furthermore, we wonder whether these amino acid sites influencing YBX1-hY4F interaction play roles in EVs sorting of hY4F. Hence, we overexpressed wild type and site-specific mutant plasmids into YBX1-KO A549 cells, and the qPCR results revealed that only the K264A mutant could significantly block YBX1-dependent sorting and secretion of hY4F into EVs (Fig. 6F, S8C). Despite that Y162 is not necessary for the soring of hY4F into EVs, the phosphorylation of YBX1-Y162 is significantly upregulated in lung cancer tissues (Fig. S8D), suggesting its potential roles in tumor genesis and progression.

To confirm the methylation of K264, we used synthetic biotin-labeled unmodified, monomethylated, dimethylated, and trimethylated YBX1-K264 peptides to screen for methylation-modified methyltransferases through peptide pulldown and LC-MS/MS experiments (Fig. 6G) [24]. Mass spectrometry results showed that three methyltransferases, SETD3, METTL13 and TRMT112, interacted with the methylated YBX1-K264 peptide and thus may be involved in the methylation of YBX1-K264 (Fig. 6H). Next, we used siRNA to knockdown these methyltransferases and then assessed the expression level of hY4F in A549 EVs. The qPCR results showed that SETD3 knockdown inhibits sorting of hY4F into A549 EVs (Fig. 6I, J), which is similar to YBX1-dependent regulation of hY4F sorting into EVs. While other two methyltransferases, METTL13 and TRMT112, are not indispensable for the sorting of EVs-hY4F (Fig. S8E-H). We investigated the presence of dimethylated modification at K264 of exogenous YBX1 protein in A549 cells using an anti-dimethylated lysine pan antibody and found that the methylation modification was significantly reduced when K264 was mutated to alanine (Fig. 6K, S8I). In addition, SETD3 knockdown significantly reduced the level of YBX1 methylation (Fig. 6L). And YBX1 methylation by SETD3 was furtherly analyzed in vitro by endogenous immunoprecipitation assay using SETD3 antibody in the presence of wild-type or K264A mutant (Fig. 6M), which confirmed that SETD3 interacted with wild-type YBX1 protein and modified the K264. And the rescue experiment indicated that YBX1 recovered the downregulated hY4F level in SETD3 knockdown-A549 cells derived EVs (Fig. 6N), suggesting YBX1 is the downstream of SETD3 regulated hY4F sorting mechanism. These data demonstrate that the YBX1 C-terminal domain is necessary for its binding to hY4F, and that K264 methylation is involved in hY4F binding and sorting into lung cancer EVs.

### SETD3 promotes lung cancer cell proliferation and migration through the methyl-YBX1/EV-hY4F pathway

**Fig. 7:**
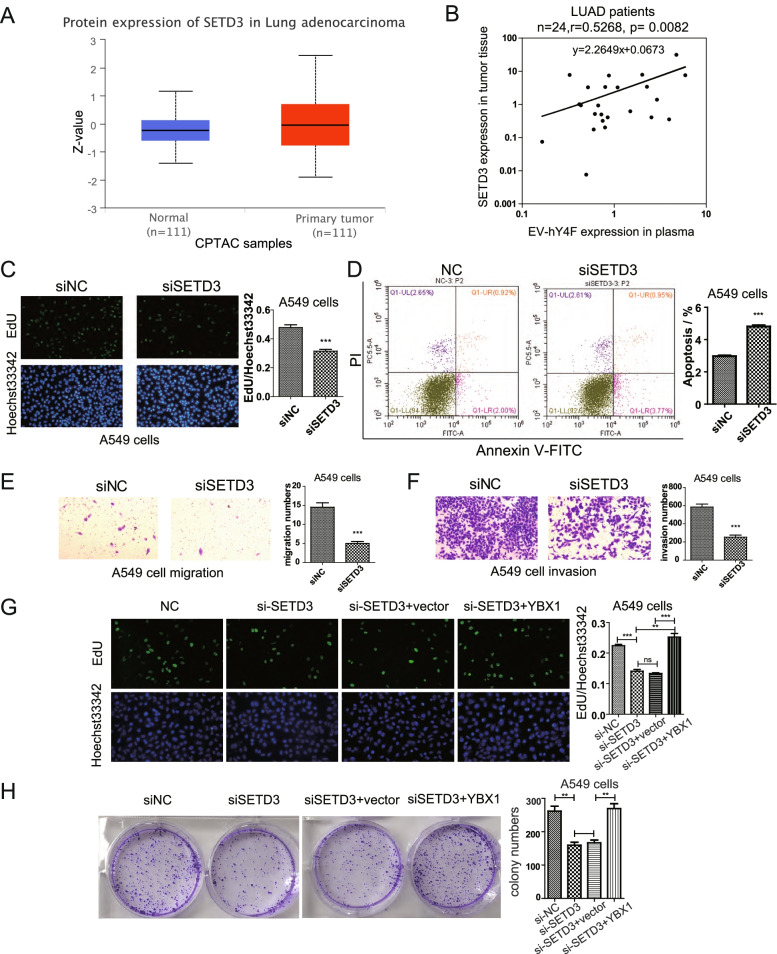
SETD3 promotes lung cancer cell proliferation and migration in A549 cells through YBX1/EVs-hY4F axis. **A** Levels of SETD3 protein in tissue from LUAD cancer patients or control subjects according to the CPTAC database (*P* = 0.0387). **B** Pearson correlation analysis of plasma EV-hY4F and tumor tissue SETD3 expression levels in lung adenocarcinoma (LUAD) patients (*n* = 24) detected by qPCR. Transcript levels of SETD3 were normalized by internal control 18S rRNA, and plasma hY4F levels were normalized by external control cel-miR-39-3p. **C** EdU assay was performed to assess the effect of SETD3 knockdown on A549 cell proliferation (at 24 h after transfection). **D** Flow cytometry using Annexin V-FITC/PI staining was performed to analyze apoptosis of A549 cells transfected with SETD3 siRNA (at 24 h after transfection). The effect of SETD3 knockdown on A549 (**E**) migration and (**F**) invasion was examined by transwell assay (at 24 h after transfection). **G** EdU assay was performed to assess the effect of YBX1 co-transfection with siRNA targeting SETD3 on A549 cells (at 48 h after transfection). **H** Colony formation assay with YBX1 and siSETD3 co-transfected A549 cells (at 7 d after transfection). Data from three independent experiments are shown as the mean ± SD (error bars). *P < 0.05, **P < 0.01, ***P < 0.001 (Student’s t-test). NC: negative control

Since the results above indicated that SETD3 regulates the sorting of hY4F into EVs, we sought to explore the biological role of SETD3 in lung cancer progression. The CPTAC database revealed that SETD3 protein was highly expressed in the tissues of lung cancer patients (*p* = 0.0387) (Fig. 7A). Furtherly, we analyzed the correlation of circulating EV-hY4F and tumor SETD3 expression using plasma and paired tumor tissue samples from lung adenocarcinoma (LUAD) patients. And the results demonstrated that tumor SETD3 expression is significantly positively related to EV-hY4F level in lung adenocarcinoma patients (Fig. 7B and Table S4). To determine whether SETD3 promotes lung cancer progression, we analyzed A549 viability, migration, invasion, and apoptosis after knockdown with SETD3-specific siRNA. Our results demonstrate that SETD3 knockdown inhibits lung cancer cell proliferation (Fig. 7C), increases the percentage of apoptotic cells (Fig. 7D), and suppresses migration and invasion (Fig. 7E, F). These effects of SETD3 knockdown were also observed in H1975 cells (Fig. S9A-D). In addition, rescue experiment indicated that YBX1 co-transfection recovered the downregulated proliferation (Fig. 7G) and colony formation (Fig. 7H) ability in SETD3 knockdown-A549 cells, suggesting YBX1 is the downstream of SETD3 mediated regulation of lung cancer cells proliferation. Taken together, these data suggest that SETD3 is involved in regulating the methyl-YBX1/EVs-hY4F pathway and plays an important role in the progression of lung cancer cells.

### YBX1 is involved in tumor growth, metastasis, and lung cancer survival rate

**Fig. 8:**
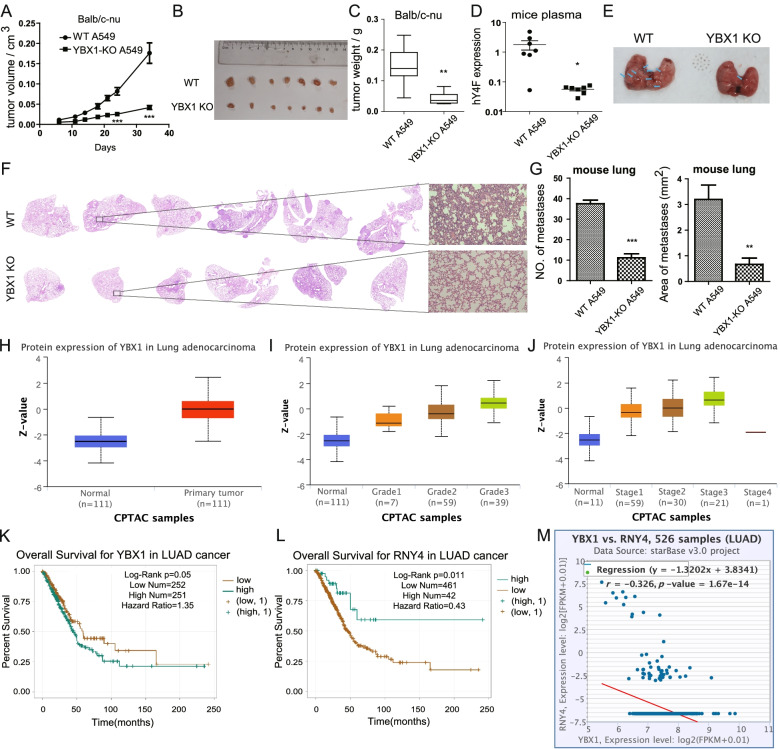
YBX1 mediated regulation of hY4F sorting into EVs is necessary for the proliferation and metastasis of lung cancer cells in vivo. **A** The growth curves of tumor volumes for xenotransplantation nude mice subcutaneously injected with WT/YBX1-KO A549 (*n* = 7). Photographs (**B**) and weights (**C**) of subcutaneous tumors at day 34 of tumor growth are shown. **D** Quantitative PCR results of hY4F in plasma from nude mice subcutaneously injected with WT/YBX1-KO A549. **E** Representative photos and (**F**) H&E stain results of lungs from nude mice injected WT/YBX1-KO A549 cells (*n* = 6) via tail vein for 5 weeks. Blue arrows indicate the tumor metastasis. **G** The statistical results of the number and area of lung metastatic lesions formed by A549 cells xenograft according to H&E stain images. **H** Levels of YBX1 protein in tissue from LUAD cancer patients or control subjects according to the CPTAC database (*p* < 0.001). The grades (**I**) and stages (**J**) of LUAD cancer patients are closely associated with YBX1 protein levels in cancer tissue according to the CPTAC database (Grade1 vs control: *p* < 0.01, Grade2/3 vs control: *p* < 0.001; Stage1/2/3 vs normal: *p* < 0.001). **K** Overall survival rate of LUAD cancer patients with high or low YBX1 mRNA level in tissue from LUAD cancer patients according to the TCGA database. **L** Overall survival rate of LUAD cancer patients with high or low hY4 RNA (RNY4) expression levels in tissue according to the TCGA database. **M** Correlational analysis of hY4F and YBX1 levels in LUAD patients according to the TCGA database. Data from three independent experiments are shown as the mean ± SD (error bars). *P < 0.05, **P < 0.01, ***P < 0.001 (Student’s t-test). WT: wild type; KO: knockout

To better understand the role of YBX1 in promoting tumor progression through hY4F in vivo, we studied the effect of subcutaneous injection of A549 cells into nude mice. Our data reveal remarkable tumor growth in these animals over 14 days. However, mice injected subcutaneously with YBX1-KO A549 cells exhibited a much slower tumor growth rate (Fig. 8A, S10). The volume and weight of lung tumors in the YBX1-KO group were significantly reduced compared to the wild type group (Fig. 8B, C). Furthermore, the hY4F levels in mouse plasma EVs were dramatically downregulated in the YBX1-KO A549 group (Fig. 8D), indicating that YBX1 is necessary for extracellular sorting of hY4F in vivo. The above results suggested that YBX1-mediated EVs sorting of hY4F plays important roles in the growth of lung cancer cells.

In addition, we established a mouse xenograft tumor metastasis model based on tail vein injection. And we found that wild-type A549 cells burdened mice had many tumors in lung surface, while YBX1-KO A549 cells burdened mice had very few tumors (Fig. 8E). And the H&E stain results showed that YBX1-KO A549 cells injected mice had fewer lung metastatic lesions than wild-type A549 injected mice (Fig. 8F). The statistical results of H&E stain indicated that YBX1-KO decreased the number and area of lung metastatic lesions formed by A549 cells xenograft (Fig. 8G).

Considering the vital function of YBX1 and hY4F in the progression of lung cancer confirmed in cell and animal levels, we wondered whether they are associated with clinical oncogenesis or prognosis of lung cancer patients. Using the CPTAC database [25], we found that YBX1 protein is significantly upregulated in lung adenocarcinoma (LUAD) patients (Fig. 8H). Consistent with this, the level of YBX1 protein in lung cancer patients correlated positively with tumor stage and grade (Fig. 8I, J). Data from the TCGA database show that YBX1 mRNA levels are closely associated with the survival rate of LUAD patients, that is, high levels of YBX1 suggest a worse prognosis (Fig. 8K). Interestingly, we found that hY4 RNA (RNY4, precursor of hY4F) levels were positively correlated with LUAD patient survival (Fig. 8L), while hY4 RNA levels were negatively correlated with YBX1 levels (Fig. 8M). The above results indicated that high expression of YBX1 is closely related to the occurrence and poor prognosis of lung cancer.

Taken together, these findings indicate that YBX1 regulates hY4F sorting into EVs in vivo, and that the accumulation of hY4F in the EVs of donor cells as a result of YBX1-KO inhibits tumor cell progression. In contrast, reduction of hY4F by YBX1-dependent sorting into EVs leads to tumor cell proliferation, migration, and invasion, eventually promoting tumor cell progression.

## Discussion

**Fig. 9:**
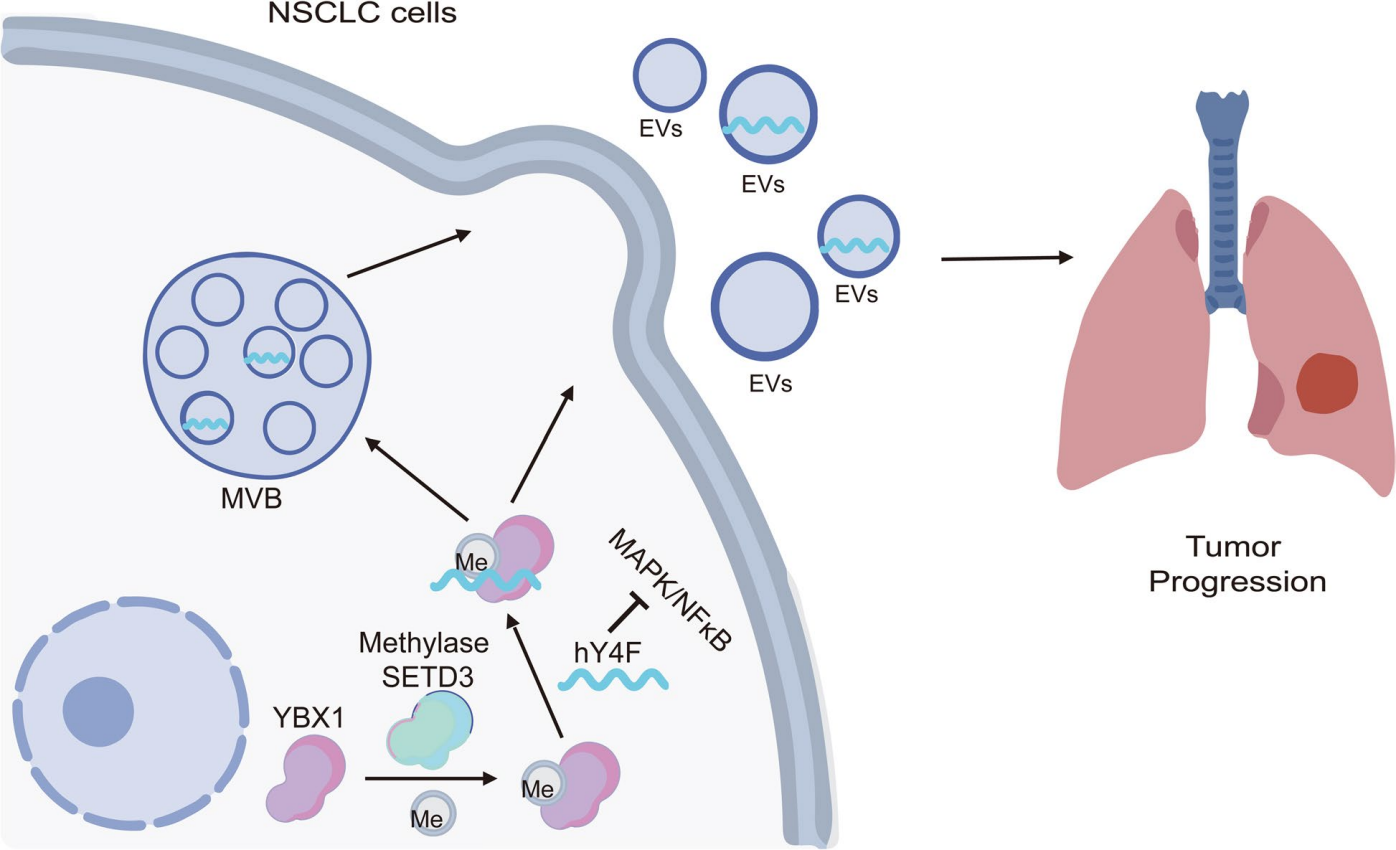
Proposed model depicting the mechanism of lung cancer progression involving the selective sorting of hY4F into EVs and regulation by methylated YBX1. The selective sorting of tumor suppressor hY4F into lung cancer EVs is regulated by interaction with RNA-binding protein YBX1. The methylation of YBX1 by the methyltransferase SETD3 affects its binding to hY4F and the subsequent secretion of hY4F into EVs, which play a role in the proliferation, migration and invasion of lung cancer cells through hY4F/MAPK/NF- κB signaling axis. MVB: multivesicular body

EVs contain a large number of ncRNAs, including YRNA fragments, which are significantly enriched in plasma EVs from lung cancer patients. EVs-derived YRNA fragments have been associated with the occurrence and development of lung cancer [26]; however, their function and mechanism in promoting tumor progression is unclear. Previously, we performed high-throughput sequencing to demonstrate that hY4F is significantly increased in plasma EVs from lung cancer patients [15]. In this paper, we confirmed that hY4F is selectively sorted into EVs by binding with YBX1 to serve as a tumor suppressor which downregulating MAPK/NF-κB signaling pathway in lung cancer. We further confirmed that the K264 lysine within the YBX1 C-terminal domain is necessary for its interaction with hY4F. Moreover, we found that this site may be modified by methylation, thus affecting the selective sorting of hY4F into EVs and progression of NSCLC (Fig. 9).

Our studies indicated that this specific type of EV-derived YRNA fragment hY4F could be a potential target for lung cancer diagnosis and prediction. Recent studies have also indicated that plasma hY4 RNA fragments can serve as a potential biomarker for the diagnosis and prognosis of some diseases. For instance, the level of plasma hY4 RNA fragments correlates with platelet function in patients with acute coronary syndrome [27]. In addition, hY4 RNA fragments could be a potential novel inflammatory marker that is used as a diagnostic/prognostic marker of multiple myeloma [28]. Collectively, our results provide evidence in support of developing hY4F-based lung cancer diagnosis and treatment drugs.

Moreover, several reports showed that various EV ncRNAs play important roles in the genesis and progress of cancer [4]. However, the function of specific ncRNAs in tumor-derived EVs varies. On the one hand, tumor-derived EVs release oncogenic miRNAs to promote the proliferation and metastasis of cancer cells by regulating the tumor microenvironment [29, 30]. However, cancer cells sort tumor-suppressed ncRNAs into EVs, which promotes the survival of cancer cells themselves and tumor development [31]. Our data demonstrate that hY4 fragments function as tumor suppressors in NSCLC genesis and progression. Unlike miRNAs, YRNA-derived fragments lack gene silencing ability [32], and relatively little is known about their signaling pathways and regulatory effects. Although the role of YRNA fragments has yet to be fully defined, they have been shown to be deregulated in many diseases, including cancer [33]. Moreover, YRNA fragments regulate cell death and inflammation in macrophages [34]. Similarly, our results suggest that EV-derived hY4F suppresses tumor progression by regulating apoptosis in lung cancer cells. And we also identified that hY4F inhibits lung cancer progression through downregulating MAPK/NF-κB signaling pathway.

Interestingly, we found hY4F is upregulated in lung cancer cell-derived EVs, but down-regulated in lung cancer cells, suggesting that hY4F is sorted into EVs in a selective manner. Many studies have indicated that selective ncRNA sorting into EVs is regulated by RNA-binding proteins [35, 36]. For example, a recent study demonstrated that hnRNPA1 regulates the packaging of miR-196a into cancer-associated fibroblast-derived exosomes by binding to its UAGGUA motif and plays an active role in head and neck cancer progression and chemoresistance [37]. Synaptotagmin-binding cytoplasmic RNA-interacting protein binds to miRNAs with a common hEXO motif (GGCU) to regulate miRNA secretion into exosomes [38]. YRNA has been shown to bind Ro60 protein, forming the Ro ribonucleoprotein complex that promotes nuclear export and maintains stability in the cytoplasm [39]. Consistent with these results, we identified YBX1 as the RNA-binding protein (RBP) of hY4F, and found that YBX1 binding promotes hY4F sorting into EVs. Although RBPs regulate the sorting of some ncRNAs into EVs in a sequence-dependent manner, the majority of ncRNAs are sorted into EVs without sequence specificity. Importantly, Sheckman and colleagues found that YBX1 protein regulates the selective sorting of small RNAs (including Y RNAs, tRNAs, miRNAs, and vault RNAs) into exosomes by binding to them without a specific motif [21]. Based on these reports, it is likely that the interaction between YBX1 and hY4F is not sequence-specific.

Investigation of the sequence and domain characteristics of YBX1 demonstrated that the C-terminal domain, not the CSD, is necessary for binding to hY4F. Interestingly, reports have shown that post-translational modification plays important roles in ncRNA sorting into EVs. For instance, SUMOylation of RNA-binding protein hnRNPA2B1 regulates its binding to miRNA, thus mediating the sorting of miRNA into EVs [23]. Therefore, we hypothesized that post-translational modification of YBX1 may be a key regulator of its binding to hY4F and the subsequent sorting of hY4F into EVs. We found that lysine K264 within the YBX1 C-terminal domain is necessary for its interaction with hY4F and confirmed that the methylation of lysine K264 is vital for the secretion of hY4F into EVs.

## Conclusions

Collectively, we revealed the role of EV-derived YRNA fragments, such as hY4F, in tumor progression. Our studies demonstrate a novel mechanism in which lysine methylation of YBX1 regulates the sorting of hY4F into EVs. Components of the methyl-YBX1-EVs-hY4F/MAPK/NF-κB signaling axis may serve as potential biomarkers and targets for cancer diagnosis and treatment.

## Supplementary Information

**Additional file 1: Figure S1.** hY4 RNA fragments inhibit the progress of lung cancer. (A) The level of hY4F RNA in plasma from healthy control subjects and lung cancer patients with different histology or stage was assessed by qPCR. CTRL (healthy control): *n* = 34; LUAD (lung adenocarcinoma): *n* = 25; LUSC (lung squamous carcinoma): *n* = 20; NSCLC stage III: *n* = 10; NSCLC stage IV: *n* = 16. (B) The hY4F level in EVs and cell lysates from hY4F mimic/NC-transfected H1975 cells was measured by qPCR (at 48 h after transfection). (C) CCK-8 (at 24, 48, and 72 h after transfection) and (D) EdU assays (at 48 h after transfection) were performed to examine the effect of a hY4F mimic on H1975 cell proliferation. (E) Flow cytometry using Annexin V-FITC/PI staining was performed to analyze apoptosis of hY4F-overexpressing H1975 cells (at 24 h after transfection). (F-G) Influences of hY4F mimic on the migration and invasion of H1975 cells were detected by Transwell assay (at 24 h after transfection). Data from three independent experiments are shown as the mean ± SD (error bars). Ns: no significance. *P < 0.05, **P < 0.01, ***P < 0.001 (Student’s t-test). NC: negative control.

**Additional file 2: Figure S2.** Knockdown of hY4 RNA fragments inhibits proliferation of IMR-90 cells. (A) Knockdown efficiency of siRNA target hY4F in IMR-90 cells was analyzed by qPCR (at 48 h after transfection). (B) EdU assay was performed to examine the effect of a siRNA target hY4F on IMR-90 cell proliferation (at 48 h after transfection).

**Additional file 3: Figure S3.** Selective sorting of hY4F into EVs inhibits the proliferation, migration, and invasion of lung cancer cells. (A) H1975 cells were incubated with hY4F mimic/NC-overexpressing A549 EVs for 48 h and then the level of hY4F was examined by qPCR. (B) The EdU assay was performed to assess the effect of hY4F-overexpressing A549 EVs on H1975 cell proliferation (at 48 h after treatment). (C) Flow cytometry using Annexin V-FITC/PI staining was performed on H1975 cells incubated with hY4F-enriched A549 EVs to analyze apoptosis (at 48 h after treatment). (D-E) The effect of hY4F-enriched A549 EVs on H1975 migration and invasion were assessed by transwell assay (at 48 h after treatment). Data from three independent experiments are shown as the mean ± SD (error bars). *P < 0.05, **P < 0.01, ***P < 0.001 (Student’s t-test). NC: negative control.

**Additional file 4: Figure S4.** DEGs enriched into MAPK pathway. Five coding genes DUSP1 (MKP), NA4A1 (Nur77), JUN (AP1 subunit), FOS (c-FOS), and CHUK (IKK) downregulated by hY4F were enriched into MAPK pathway (https://www.kegg.jp/pathway/map04010).

**Additional file 5: Figure S5.** HY4F enriched lung cancer EVs promotes proliferation and oncogenes expression in IMR-90 cells. (A) Levels of hY4F in IMR-90 cells treated with hY4F mimic/NC transfected A549 EVs detected by qPCR (at 48 h after treatment). (B) EdU assay performed to assess the effect of hY4F-enriched A549 EVs on IMR-90 cells proliferation (at 48 h after treatment). (C) QRT-PCR analysis results of the MAPK pathway related genes in IMR-90 cells incubated with hY4F-A549 EVs/NC.

**Additional file 6: Figure S6.** YBX1 protein binds hY4F to regulate its sorting into lung cancer EVs. (A) Western blot analysis of hY4F binding proteins enriched by RNA pulldown assay. (B) Quantitative PCR results of hY4F in EVs and cell lysates from H1975 cells transfected with siRNA (#1) targeting YBX1. (C) Screening of YBX1 knockout A549 cells by western blot using YBX1 antibody. (D) Nanosight analysis of sizes between WT and YBX1-KO A549 cells derived EVs. (E) Quantitative PCR results of hY1F, hY3F, and hY5F in EVs and cell lysates from wild type (WT) and YBX1 knockout (KO) A549 cells. (F) Relative abundance of different YRNA fragments in both WT and YBX1-KO A549 cells. Data from three independent experiments are shown as the mean ± SD (error bars). *P < 0.05, **P < 0.01, ***P < 0.001, ns: no significant difference (Student’s t-test).

**Additional file 7: Figure S7.** YBX1 promotes proliferation and migration of lung cancer cells. (A) CCK-8 (at 24, 48, 72 and 96 h after transfection) and (B) EdU assays (at 48 h after transfection) were performed to assess the effect of YBX1 overexpression and knockdown on H1975 cell proliferation. (C) Flow cytometry using Annexin V-FITC/PI staining was performed to analyze apoptosis of H1975 cells transfected with YBX1 plasmid or siRNA (at 24 h after transfection). The effect of YBX1 overexpression and knockdown on H1975 (D) migration and (E) invasion was examined by Transwell assay (at 24 h after transfection). Data from three independent experiments are shown as the mean ± SD (error bars). *P < 0.05, **P < 0.01, ***P < 0.001 (Student’s t-test). NC: negative control.

**Additional file 8: Figure S8.** Screening for post-translational modification of YBX1 and potential roles of methyltransferase on EV secretion of hY4F. (A-B) Biotin-labelled hY4F RNA pulldown analysis of cell lysates harvested from A549 overexpressing FLAG-tagged wild type (WT) or mutant YBX1. The K/Q, K/R and K/M mutation is used for simulating acetylated, deacetylated and methylated lysine. (C) Quantitative PCR data of hY4F in EVs from YBX1-KO A549 cells transfected with wild type (WT) or mutant YBX1. (D) Levels of phosphorylated Y162 modified YBX1 protein in tissue from LUAD cancer patients or control subjects according to the CPTAC database. (*p*<0.001) (E) Quantitative PCR data of hY4F in EVs and cell lysates from A549 cells transfected with METTL13 siRNA and its knockdown effect were analyzed. (F) The knockdown effect of siRNA targeting METTL13 was examined by qRT-PCR. (G) Quantitative PCR data of hY4F in EVs and cell lysates from A549 cells transfected with TRMT112 siRNA. (H) The knockdown effect of siRNA targeting TRMT112 was examined by qRT-PCR. (I) Dot blot assay to identify the specificity of di-methyl-lysine pan antibody. Data from three independent experiments are shown as the mean ± SD (error bars). *P < 0.05, **P < 0.01, ***P < 0.001 (Student’s t-test). NC: negative control.

**Additional file 9: Figure S9.** SETD3 is necessary for the proliferation and migration of lung cancer cells. (A) EdU assay was performed to assess the effect of SETD3 knockdown on H1975 cell proliferation (at 48 h after transfection). (B) Flow cytometry using Annexin V-FITC/PI staining was performed to analyze apoptosis of H1975 cells transfected with YBX1 siRNA (at 24 h after transfection). (C-D) The effect of SETD3 knockdown on H1975 migration and invasion was examined by Transwell assay (at 24 h after transfection). Data from three independent experiments are shown as the mean ± SD (error bars). *P < 0.05, **P < 0.01, ***P < 0.001 (Student’s t-test). NC: negative control.

**Additional file 10: Figure S10.** Photograph of mice subcutaneously injected with WT/YBX1-KO A549 cells after 34 days.

**Additional file 11: Table S1.** Clinical characteristics of plasma samples from NSCLC patients and healthy controls.

**Additional file 12: Table S2.** Differential expressed genes responded to hY4F overexpression in A549 cells identified by mRNA sequencing.

**Additional file 13: Table S3.** Mass spectrometric analysis of potential hY4F RNA-binding proteins following RNA pulldown assay.

**Additional file 14: Table S4.** Clinical characteristics of tissues and paired plasma from lung adenocarcinoma (LUAD) patients.

## Data Availability

The data supporting the conclusions of this article are presented within the article and its additional files. The datasets generated and analyzed during the current study are available in TCGA (https://cancergenome.nih.gov/) and CPTAC (https://proteomics.cancer.gov/programs/cptac).

## Abbreviations

EVs: Extracellular vesicles
NSCLC: Non-small cell lung cancer
YBX1: Y box binding protein 1
hY4F: hY4 RNA fragment
qRT-PCR: Quantitative reverse transcription polymerase chain reaction
ncRNA: Non-coding RNAs
RoRNPs: Ro ribonucleoproteins
LC-MS/MS: Liquid chromatography-tandem mass spectrometry
LUAD: Lung adenocarcinoma
SETD3: SET domain containing 3
SD: Standard deviation
CCK-8: Cholecystokinin octapeptide
TCGA: The Cancer Genome Atlas
CPTAC: Clinical Proteomic Tumor Analysis Consortium
