# Supplementary figures and images for: Selective sorting and secretion of hY4 RNA fragments into extracellular vesicles mediated by methylated YBX1 to promote lung cancer progression
Source: J Exp Clin Cancer Res. 2022 Apr 11;41:136. doi: 10.1186/s13046-022-02346-w (PMC8996536; doi:10.1186/s13046-022-02346-w)

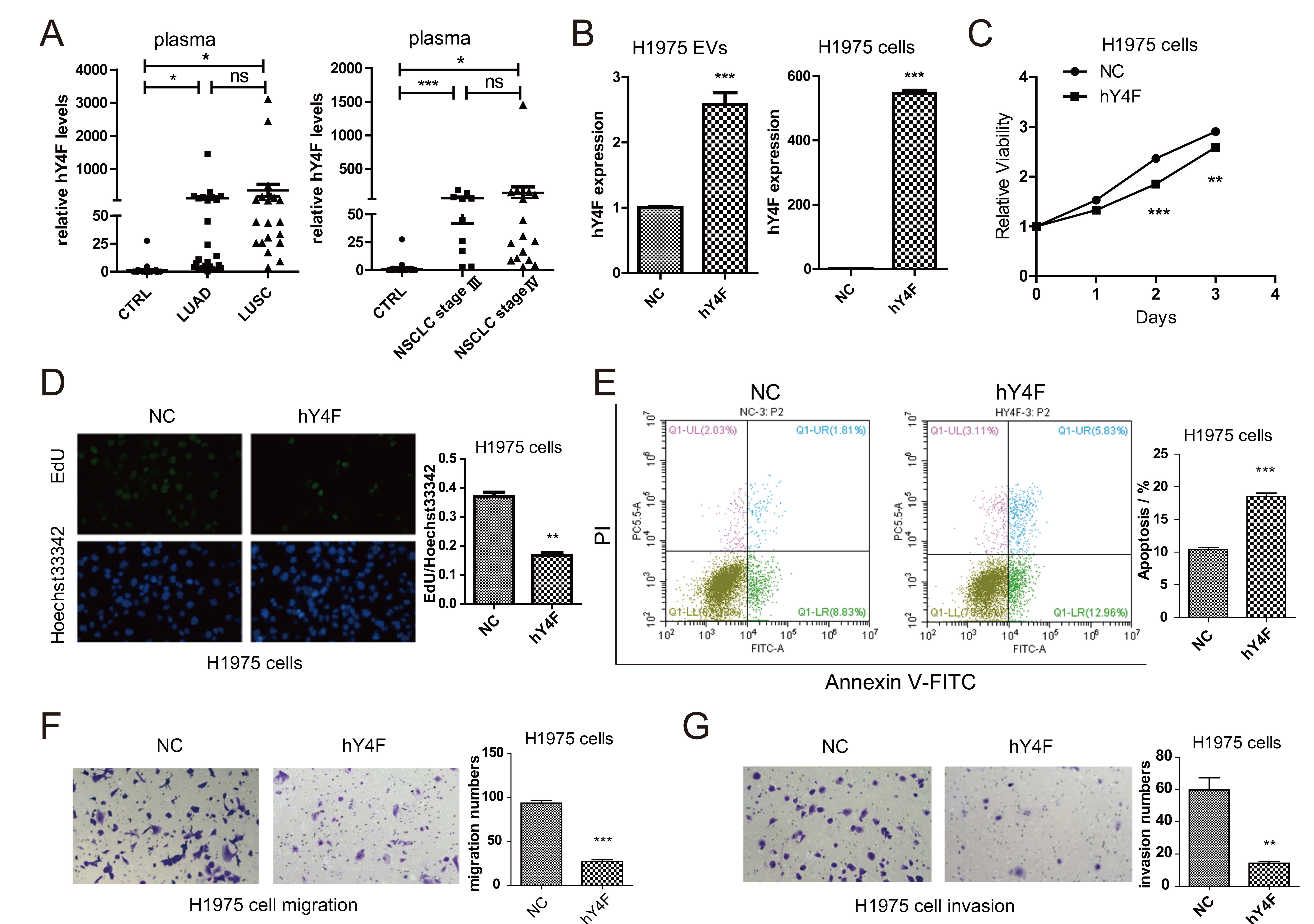

Supplement: Supplementary file 1 — Additional file 1: Figure S1. hY4 RNA fragments inhibit the progress of lung cancer. (A) The level of hY4F RNA in plasma from healthy control subjects and lung cancer patients with different histology or stage was assessed by qPCR. CTRL (healthy control): n = 34; LUAD (lung adenocarcinoma): n = 25; LUSC (lung squamous carcinoma): n = 20; NSCLC stage III: n = 10; NSCLC stage IV: n = 16. (B) The hY4F level in EVs and cell lysates from hY4F mimic/NC-transfected H1975 cells was measured by qPCR (at 48 h after transfection). (C) CCK-8 (at 24, 48, and 72 h after transfection) and (D) EdU assays (at 48 h after transfection) were performed to examine the effect of a hY4F mimic on H1975 cell proliferation. (E) Flow cytometry using Annexin V-FITC/PI staining was performed to analyze apoptosis of hY4F-overexpressing H1975 cells (at 24 h after transfection). (F-G) Influences of hY4F mimic on the migration and invasion of H1975 cells were detected by Transwell assay (at 24 h after transfection). Data from three independent experiments are shown as the mean ± SD (error bars). Ns: no significance. *P < 0.05, **P < 0.01, ***P < 0.001 (Student’s t-test). NC: negative control. [file 13046_2022_2346_MOESM1_ESM.tif]

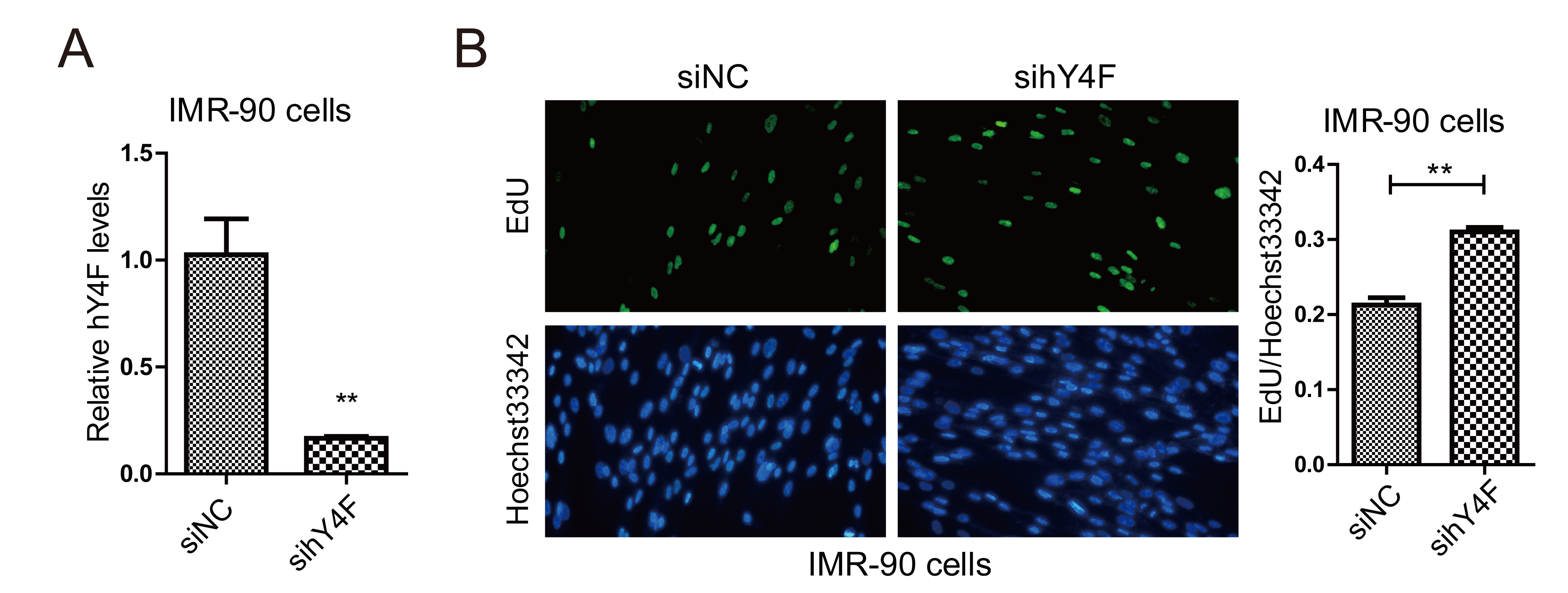

Supplement: Supplementary file 2 — Additional file 2: Figure S2. Knockdown of hY4 RNA fragments inhibits proliferation of IMR-90 cells. (A) Knockdown efficiency of siRNA target hY4F in IMR-90 cells was analyzed by qPCR (at 48 h after transfection). (B) EdU assay was performed to examine the effect of a siRNA target hY4F on IMR-90 cell proliferation (at 48 h after transfection). [file 13046_2022_2346_MOESM2_ESM.tif]

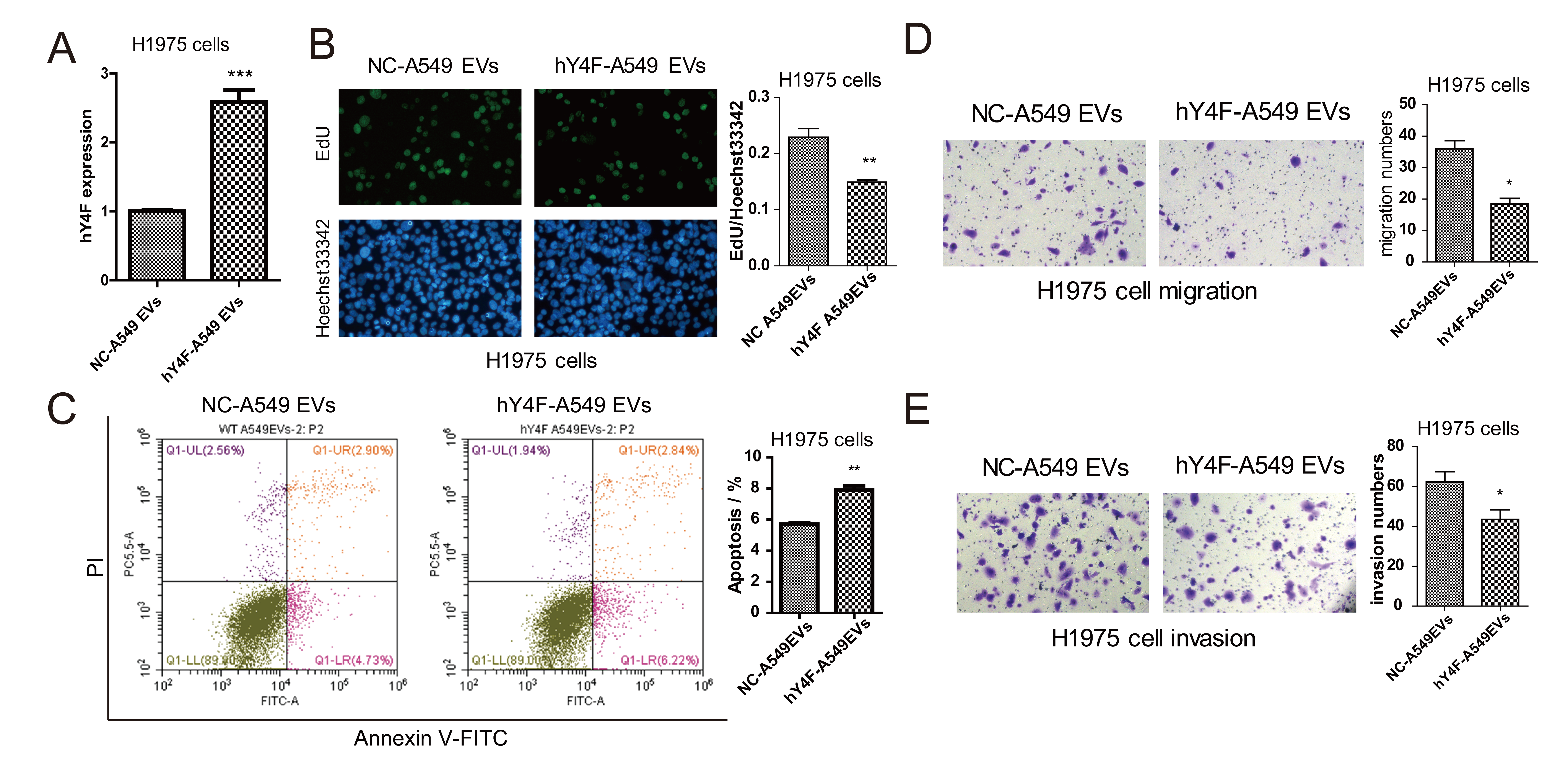

Supplement: Supplementary file 3 — Additional file 3: Figure S3. Selective sorting of hY4F into EVs inhibits the proliferation, migration, and invasion of lung cancer cells. (A) H1975 cells were incubated with hY4F mimic/NC-overexpressing A549 EVs for 48 h and then the level of hY4F was examined by qPCR. (B) The EdU assay was performed to assess the effect of hY4F-overexpressing A549 EVs on H1975 cell proliferation (at 48 h after treatment). (C) Flow cytometry using Annexin V-FITC/PI staining was performed on H1975 cells incubated with hY4F-enriched A549 EVs to analyze apoptosis (at 48 h after treatment). (D-E) The effect of hY4F-enriched A549 EVs on H1975 migration and invasion were assessed by transwell assay (at 48 h after treatment). Data from three independent experiments are shown as the mean ± SD (error bars). *P < 0.05, **P < 0.01, ***P < 0.001 (Student’s t-test). NC: negative control. [file 13046_2022_2346_MOESM3_ESM.tif]

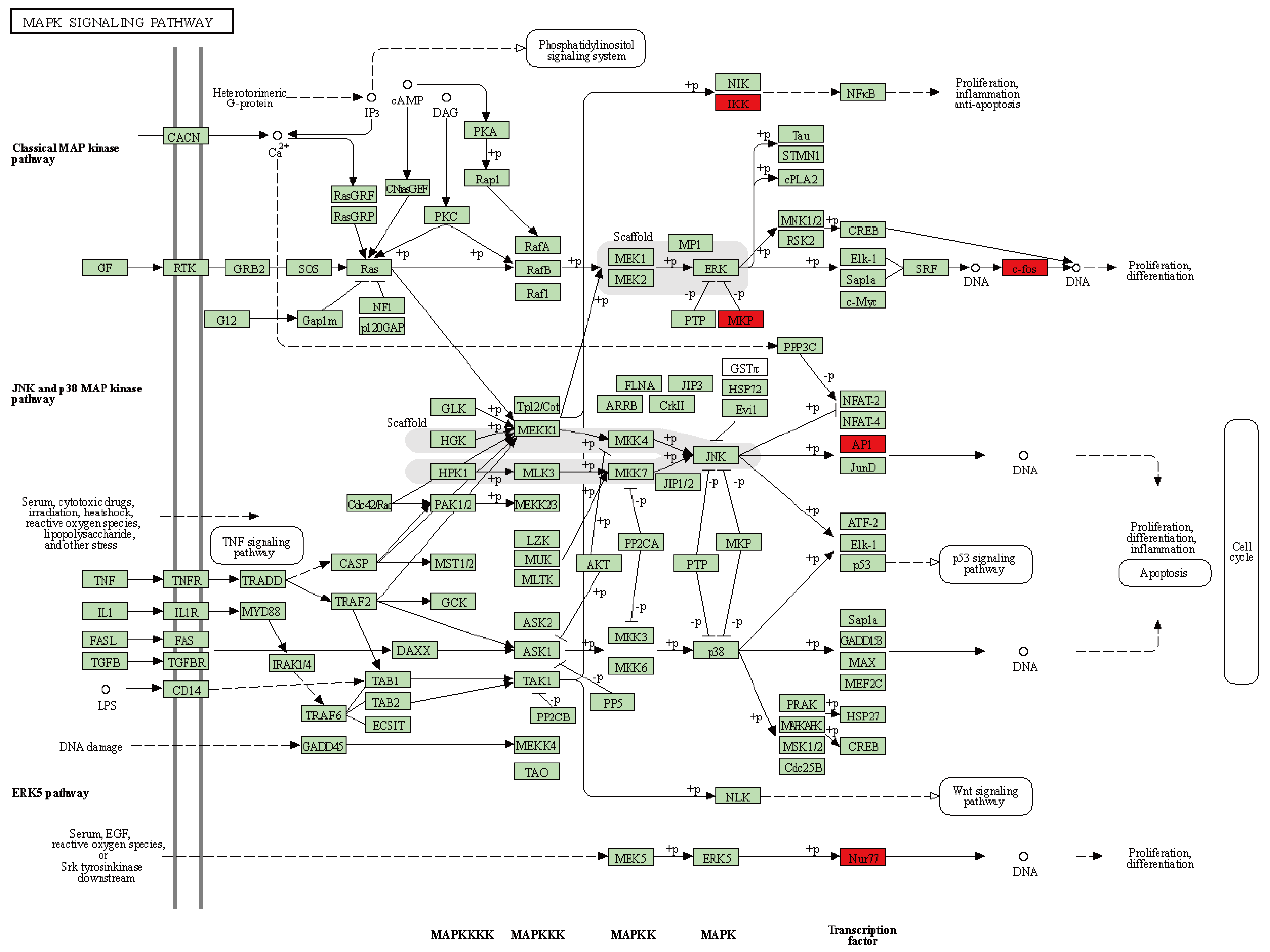

Supplement: Supplementary file 4 — Additional file 4: Figure S4. DEGs enriched into MAPK pathway. Five coding genes DUSP1 (MKP), NA4A1 (Nur77), JUN (AP1 subunit), FOS (c-FOS), and CHUK (IKK) downregulated by hY4F were enriched into MAPK pathway (https://www.kegg.jp/pathway/map04010). [file 13046_2022_2346_MOESM4_ESM.tif]

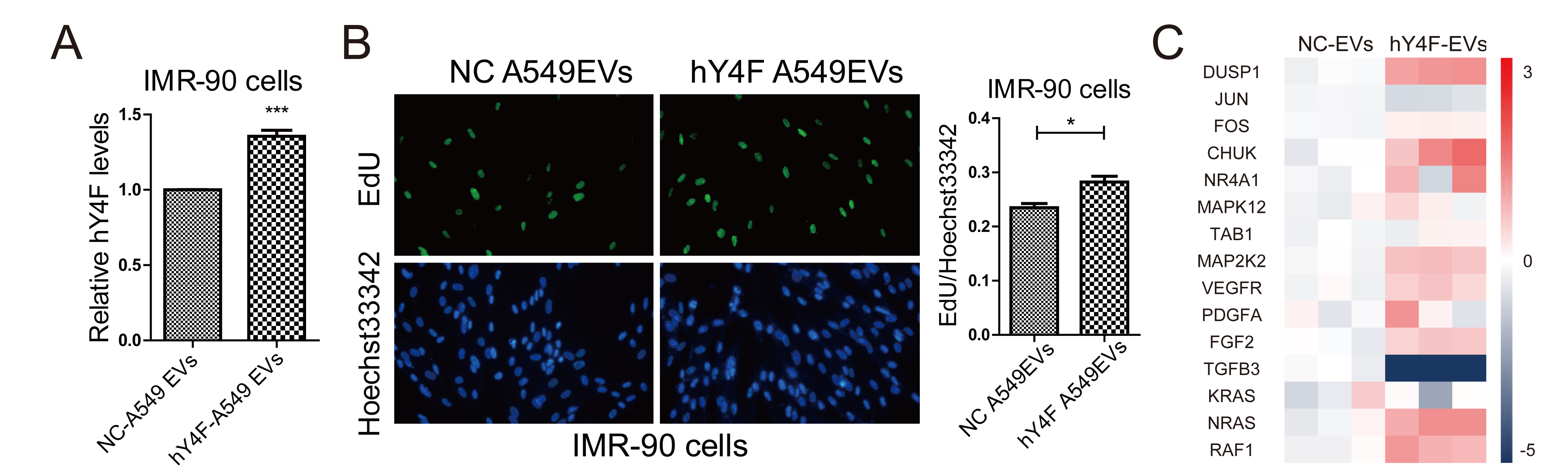

Supplement: Supplementary file 5 — Additional file 5: Figure S5. HY4F enriched lung cancer EVs promotes proliferation and oncogenes expression in IMR-90 cells. (A) Levels of hY4F in IMR-90 cells treated with hY4F mimic/NC transfected A549 EVs detected by qPCR (at 48 h after treatment). (B) EdU assay performed to assess the effect of hY4F-enriched A549 EVs on IMR-90 cells proliferation (at 48 h after treatment). (C) QRT-PCR analysis results of the MAPK pathway related genes in IMR-90 cells incubated with hY4F-A549 EVs/NC. [file 13046_2022_2346_MOESM5_ESM.tif]

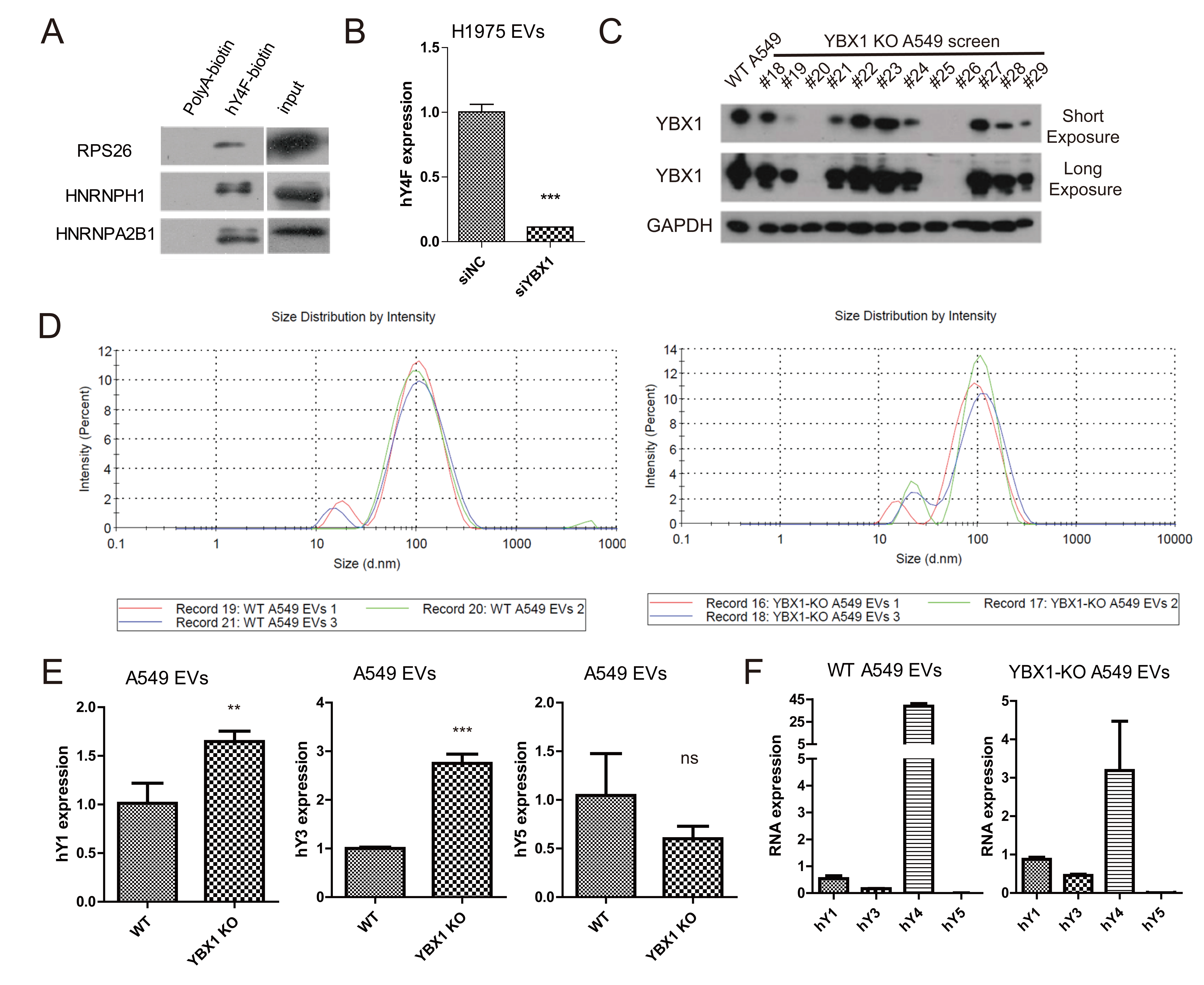

Supplement: Supplementary file 6 — Additional file 6: Figure S6. YBX1 protein binds hY4F to regulate its sorting into lung cancer EVs. (A) Western blot analysis of hY4F binding proteins enriched by RNA pulldown assay. (B) Quantitative PCR results of hY4F in EVs and cell lysates from H1975 cells transfected with siRNA (#1) targeting YBX1. (C) Screening of YBX1 knockout A549 cells by western blot using YBX1 antibody. (D) Nanosight analysis of sizes between WT and YBX1-KO A549 cells derived EVs. (E) Quantitative PCR results of hY1F, hY3F, and hY5F in EVs and cell lysates from wild type (WT) and YBX1 knockout (KO) A549 cells. (F) Relative abundance of different YRNA fragments in both WT and YBX1-KO A549 cells. Data from three independent experiments are shown as the mean ± SD (error bars). *P < 0.05, **P < 0.01, ***P < 0.001, ns: no significant difference (Student’s t-test). [file 13046_2022_2346_MOESM6_ESM.tif]

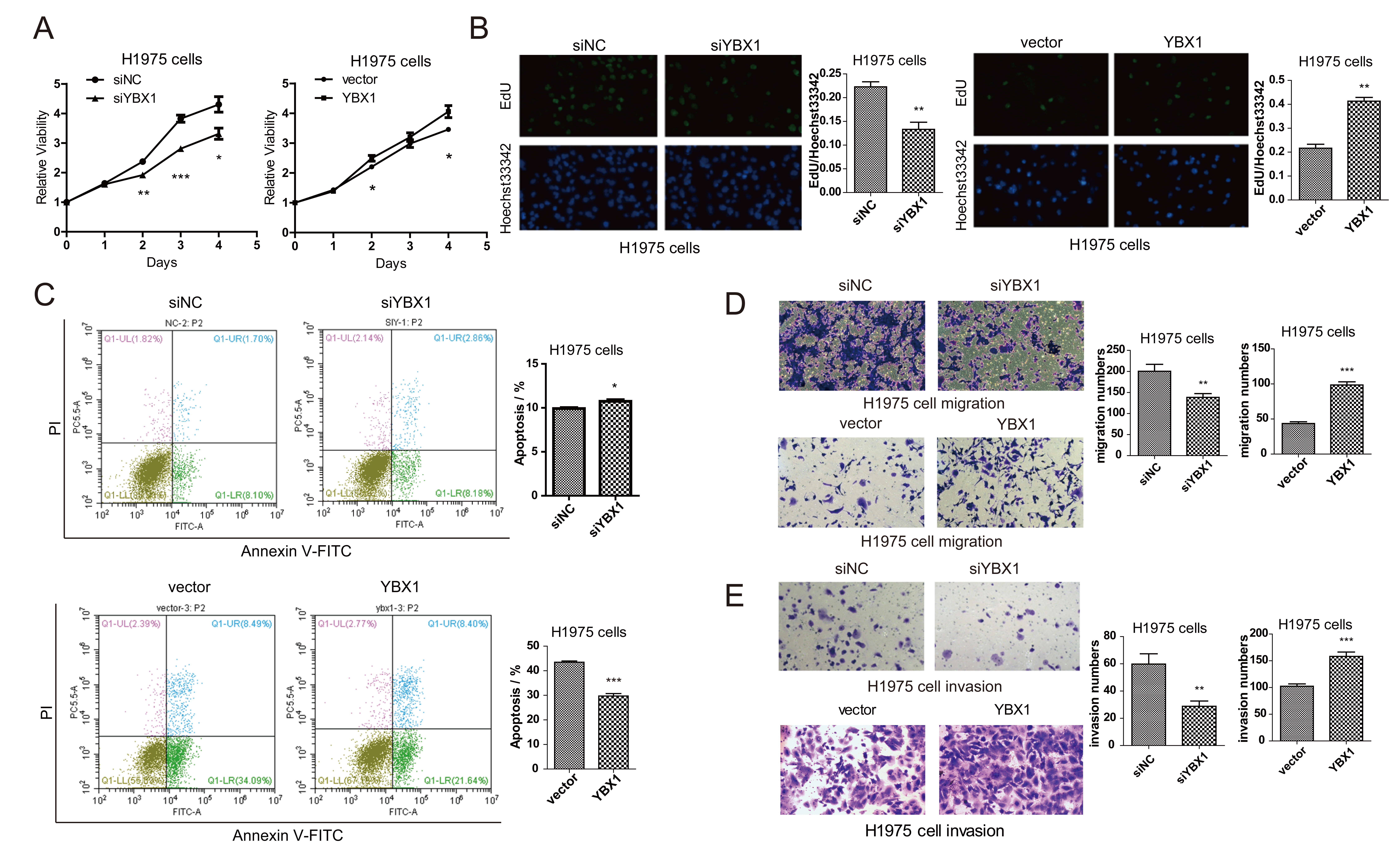

Supplement: Supplementary file 7 — Additional file 7: Figure S7. YBX1 promotes proliferation and migration of lung cancer cells. (A) CCK-8 (at 24, 48, 72 and 96 h after transfection) and (B) EdU assays (at 48 h after transfection) were performed to assess the effect of YBX1 overexpression and knockdown on H1975 cell proliferation. (C) Flow cytometry using Annexin V-FITC/PI staining was performed to analyze apoptosis of H1975 cells transfected with YBX1 plasmid or siRNA (at 24 h after transfection). The effect of YBX1 overexpression and knockdown on H1975 (D) migration and (E) invasion was examined by Transwell assay (at 24 h after transfection). Data from three independent experiments are shown as the mean ± SD (error bars). *P < 0.05, **P < 0.01, ***P < 0.001 (Student’s t-test). NC: negative control. [file 13046_2022_2346_MOESM7_ESM.tif]

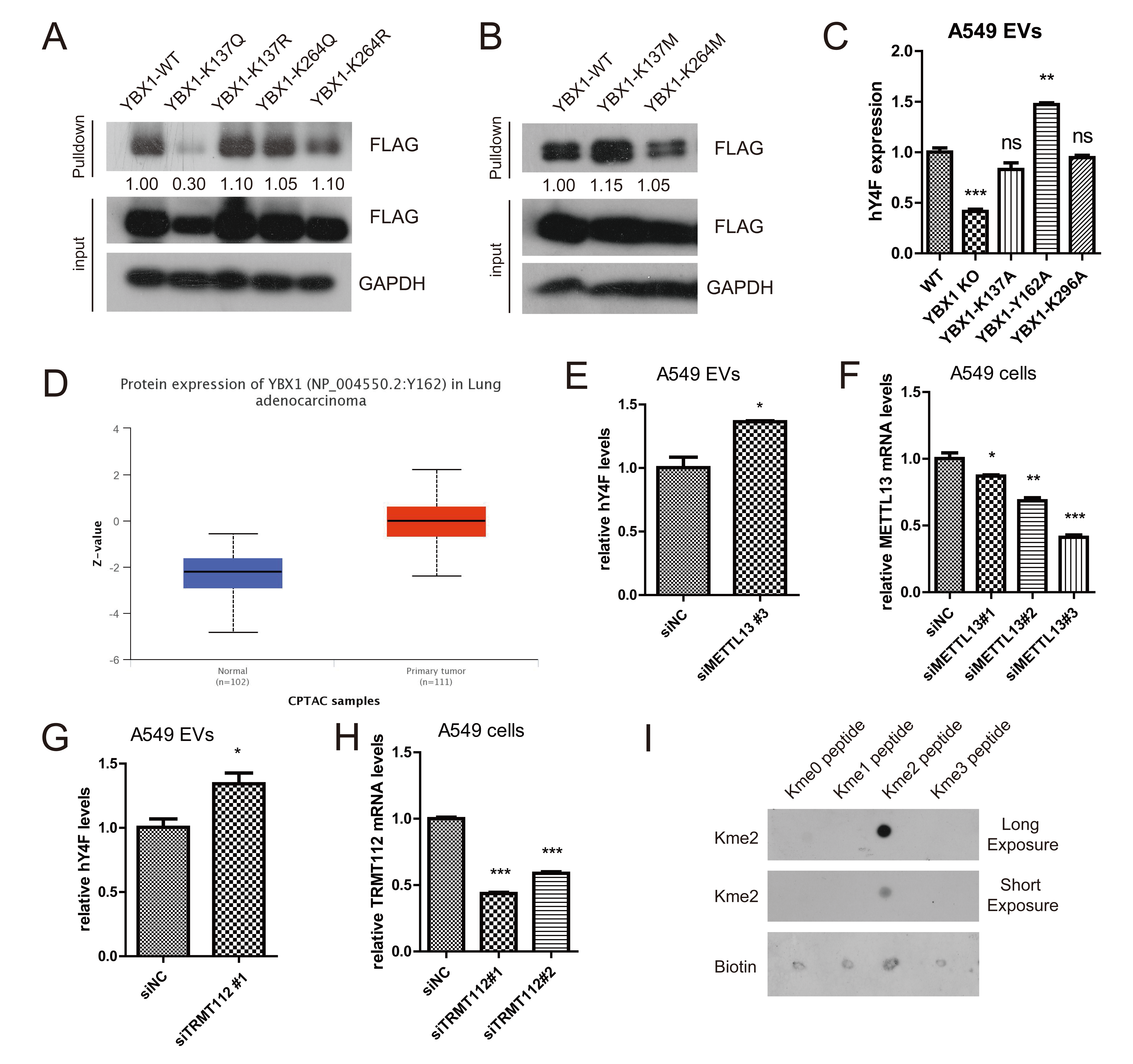

Supplement: Supplementary file 8 — Additional file 8: Figure S8. Screening for post-translational modification of YBX1 and potential roles of methyltransferase on EV secretion of hY4F. (A-B) Biotin-labelled hY4F RNA pulldown analysis of cell lysates harvested from A549 overexpressing FLAG-tagged wild type (WT) or mutant YBX1. The K/Q, K/R and K/M mutation is used for simulating acetylated, deacetylated and methylated lysine. (C) Quantitative PCR data of hY4F in EVs from YBX1-KO A549 cells transfected with wild type (WT) or mutant YBX1. (D) Levels of phosphorylated Y162 modified YBX1 protein in tissue from LUAD cancer patients or control subjects according to the CPTAC database. (p<0.001) (E) Quantitative PCR data of hY4F in EVs and cell lysates from A549 cells transfected with METTL13 siRNA and its knockdown effect were analyzed. (F) The knockdown effect of siRNA targeting METTL13 was examined by qRT-PCR. (G) Quantitative PCR data of hY4F in EVs and cell lysates from A549 cells transfected with TRMT112 siRNA. (H) The knockdown effect of siRNA targeting TRMT112 was examined by qRT-PCR. (I) Dot blot assay to identify the specificity of di-methyl-lysine pan antibody. Data from three independent experiments are shown as the mean ± SD (error bars). *P < 0.05, **P < 0.01, ***P < 0.001 (Student’s t-test). NC: negative control. [file 13046_2022_2346_MOESM8_ESM.tif]

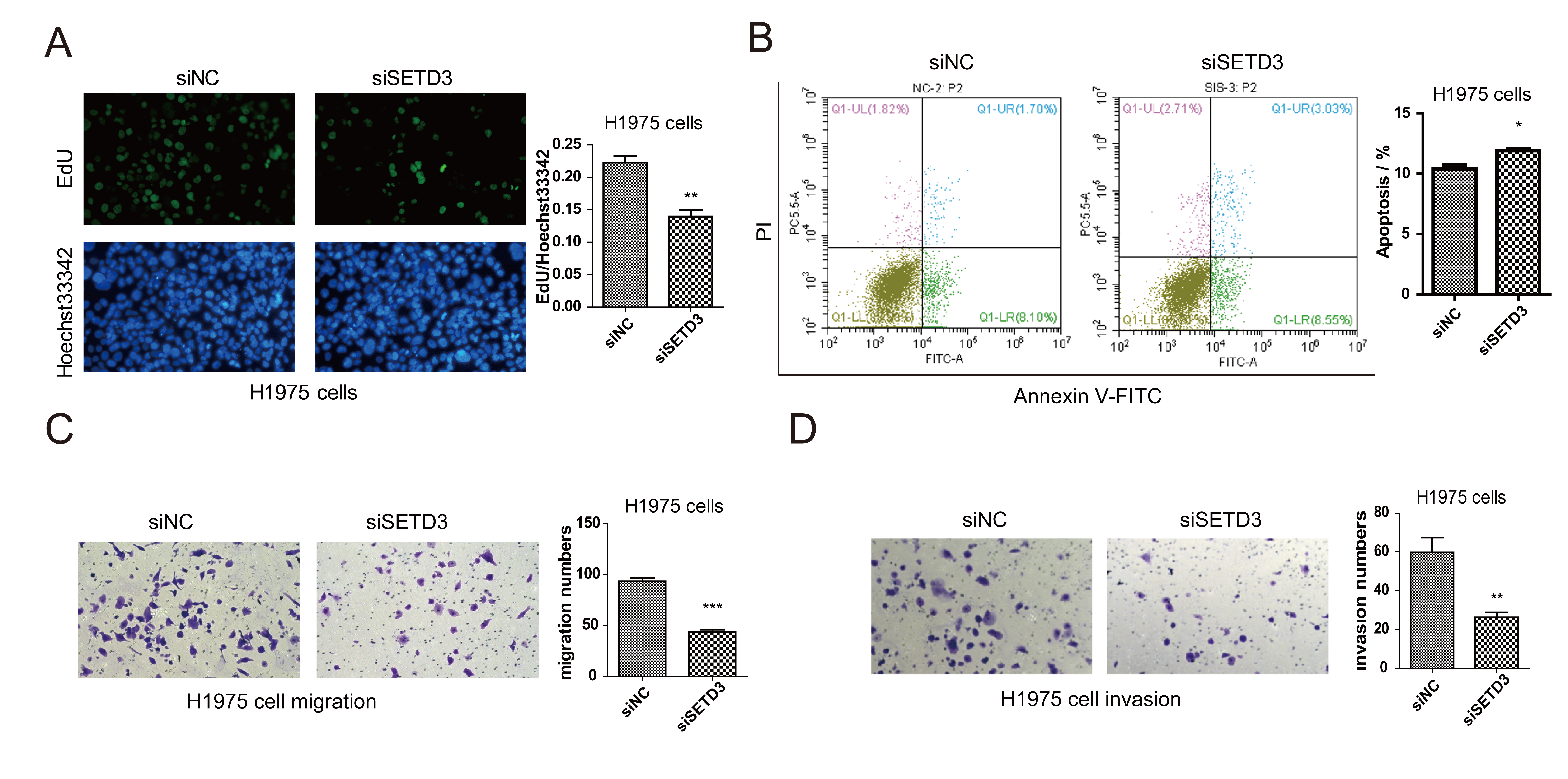

Supplement: Supplementary file 9 — Additional file 9: Figure S9. SETD3 is necessary for the proliferation and migration of lung cancer cells. (A) EdU assay was performed to assess the effect of SETD3 knockdown on H1975 cell proliferation (at 48 h after transfection). (B) Flow cytometry using Annexin V-FITC/PI staining was performed to analyze apoptosis of H1975 cells transfected with YBX1 siRNA (at 24 h after transfection). (C-D) The effect of SETD3 knockdown on H1975 migration and invasion was examined by Transwell assay (at 24 h after transfection). Data from three independent experiments are shown as the mean ± SD (error bars). *P < 0.05, **P < 0.01, ***P < 0.001 (Student’s t-test). NC: negative control. [file 13046_2022_2346_MOESM9_ESM.tif]

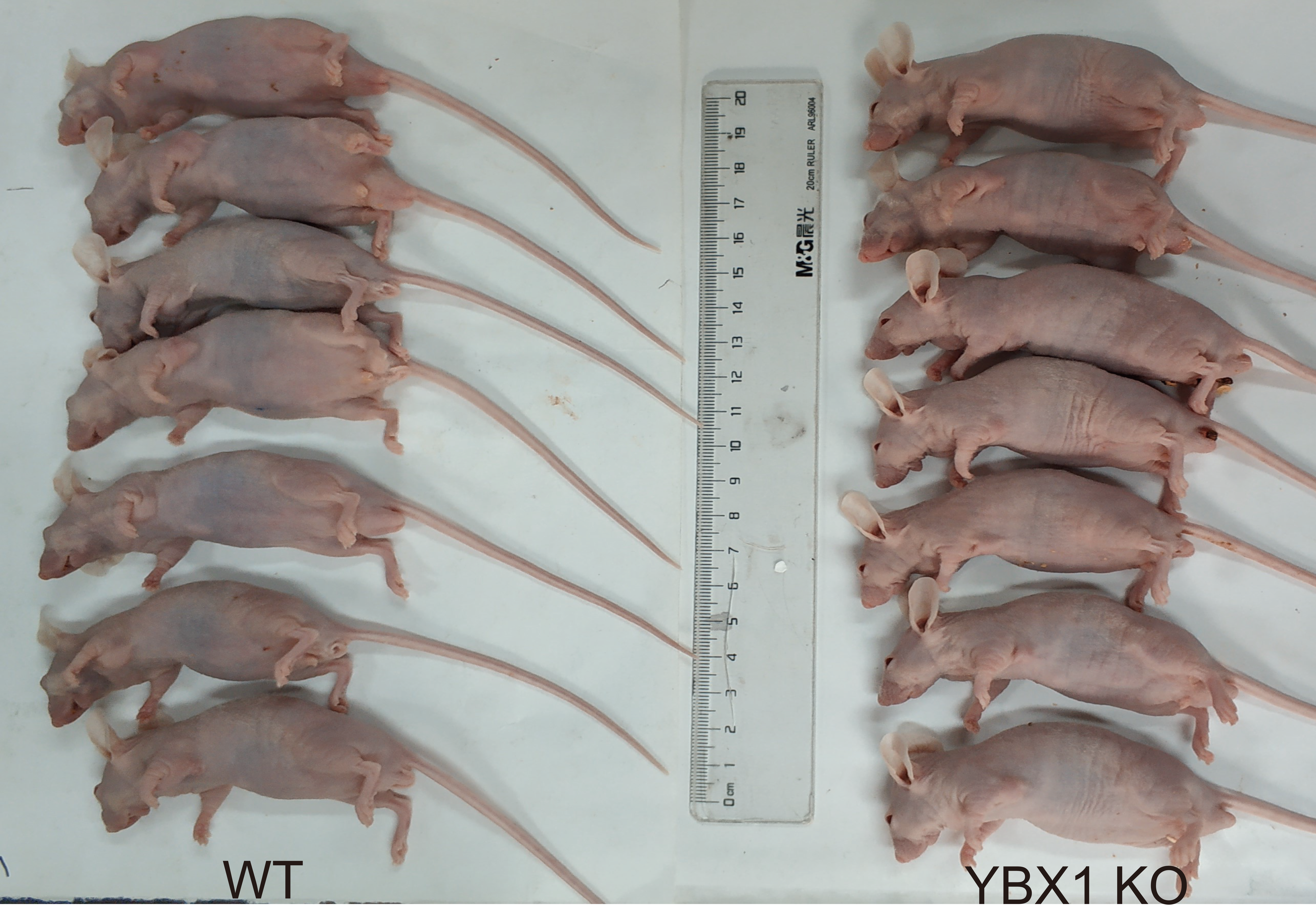

Supplement: Supplementary file 10 — Additional file 10: Figure S10. Photograph of mice subcutaneously injected with WT/YBX1-KO A549 cells after 34 days. [file 13046_2022_2346_MOESM10_ESM.tif]
